# Robust evidence for theta-band rhythmicity in behavior across two dense-sampling datasets

**DOI:** 10.1038/s44271-026-00500-0

**Published:** 2026-07-07

**Authors:** Mengting Xu, Esperanza Badaya, Mehdi Senoussi, Tom Verguts

**Affiliations:** 1 Department of Experimental Psychology, Faculty of Psychology and Educational Sciences, Ghent University, Ghent, Belgium; 2 Univ Toulouse, Université Toulouse Jean Jaurès, CLLE, CNRS, Toulouse, France

**Keywords:** Human behaviour, Cognitive control

## Abstract

Rhythmic fluctuations in neuronal excitability, called neural oscillations, pervade brain activity. If these oscillations have a functional role in behavior, they should also be expressed in a behavioral signature. However, identifying such behavioral signatures remains challenging and continues to motivate debate, particularly because aperiodic temporal structure can sometimes be mistaken for rhythmic activity. To assess behavioral rhythmicity while controlling for aperiodic dynamics, we applied a state-of-the-art autoregressive modelling approach to two datasets: one published dataset (final sample: *n* = 34), and a new dataset (final sample: *n* = 26) collected with an adapted dense-sampling design. In both datasets, AR modelling recovered reliable behavioral oscillations in the theta band. Reanalyses of the published dataset also reproduced the reported theta shift with task demands, with more difficult tasks showing slower theta. In contrast, despite clear rhythmicity, the new dataset showed no consistent frequency shift across conditions. Overall, this study establishes rigorous and robust detection of behavioral oscillations. It also suggests that the frequency modulation of behavioral oscillations by task difficulty requires further study.

## Abstract (web summary)

This study asks whether cognition unfolds rhythmically. Across two dense-sampling datasets, autoregressive modelling reveals reliable theta-band rhythms in behavior, but no consistent difficulty-related frequency shift.

## Introduction

A central challenge for cognition is minimizing interference between concurrently processed information. One influential proposal is that the brain accomplishes this by using neural oscillations, which function as an internal clock to segregate competing representations into separate temporal windows, thereby gating information flow and reducing cross-talk^1–4^. A related challenge is how competing sensory and action-relevant information is prioritized over time. Recent theoretical accounts suggest that perception and attention may operate through dynamic fluctuations rather than continuous processing, allowing task-relevant information to be periodically prioritized while the system remains sensitive to new input^5,6^. Nonetheless, whether such oscillations are necessary or even genuinely present remains debated. Some have argued that cognitive operations (i.e., feature binding) can instead be supported by alternative, non-rhythmic dynamics such as event-like, aperiodic transients in spiking activity or more sustained changes in firing rates, without relying on oscillatory synchronization^7–9^, or that feature binding is not even necessary^10^. We contribute to this debate by providing converging evidence that is consistent with the oscillatory segregation account.

Electrophysiological studies have extensively documented how oscillatory dynamics are engaged and modulated by cognitive demands. Theta rhythms, for example, support multi-item working memory maintenance, slowing to accommodate more items^11–13^, whereas alpha rhythms often accelerate under higher cognitive load^14^. Such frequency shifts also contribute to the refined sampling of attentional^15,16^ and perceptual information^17,18^, which typically operate at distinct rhythms and adjust with task demands. This adaptability makes oscillatory frequency a promising marker of cognitive flexibility.

At a finer timescale, phase-dependent effects provide further support for this temporal account. The instantaneous phase of ongoing rhythms predicts performance in perception^19–22^, attention^21,23^, and memory^24,25^, reinforcing the view that neural computations are timed by oscillatory activity. A key question, however, follows: if this rhythmic organization is indeed fundamental to cognition, does its signature translate to overt, measurable behavior?

A growing body of research reports such periodic fluctuations in performance, known as behavioral oscillations, which provide compelling evidence for the role of neural rhythms in cognition. Indeed, if oscillations are a mere epiphenomenon of neural processing, it is unclear why or how they would have downstream effects on behavior. Studies using a dense-sampling design have revealed theta-rhythmic (~4–8 Hz) oscillations in visual working memory accuracy^26^, while others have shown that response times systematically vary with the phase of ongoing theta and beta oscillations^27^. If behavioral oscillations are functionally meaningful, their properties (e.g., power or frequency) may also adapt to task requirements.

Initial evidence for a behaviorally relevant frequency adaptation came from Senoussi et al.^28^, who revealed that behavioral theta rhythms shift toward an optimal frequency as task demands change. For example, theta frequency slowed down when task rules became more difficult. However, that study relied on spectral methods with limited power to separate true periodicities from artifacts of aperiodic dynamics. Moreover, the authors did not explicitly test for oscillations in the first place, leaving open questions about the robustness of the reported effects. This methodological gap motivates our use of a participant-level autoregressive model^29^. Its key advantage lies in explicitly isolating the periodic component by characterizing and removing each participant’s aperiodic structure at the individual level, thereby minimizing the risk of misinterpreting noise as behavioral rhythms. Moreover, compared with a group-level autoregressive model^30^, this pipeline does not assume phase alignment across participants, which also helps preserve individual rhythmic signals.

Using this more rigorous approach, we examined two datasets. In Experiment 1, we reanalyzed the published dataset from Senoussi et al.^28,31^, which allowed us to test whether behavioral theta oscillations remain robust after controlling for aperiodic structure, and whether their frequency still varies with task difficulty. This reanalysis not only offers direct evidence for rhythmicity in behavior but also verifies the previously reported shifts in theta frequency. In Experiment 2, we aimed to replicate this result while also addressing a limitation of the original paradigm. In that paradigm, the observed theta shift could reflect the influence of cue ambiguity or cue entropy (i.e., some cues contained two different symbols, whereas others contained one), rather than being driven by task demand. Therefore, in Experiment 2, we adapted the paradigm so that each cue consisted of two different stimuli, while keeping the cue meanings the same as in Experiment 1. Thus, we were able to dissociate cue ambiguity or entropy from task difficulty.

Together, these experiments pursue two main objectives: first, to provide stronger, methodologically robust evidence that behavior itself is rhythmic; and second, to test whether the frequency of behavioral oscillations is a general and reliable marker of cognitive demand. This is both a stringent test of the reliability of behavioral time courses and a deeper look at how the brain uses internal rhythms to organize behavior.

## Methods

The analyses reported in this study were not preregistered.

### Experiment 1

#### Participants

Thirty-nine healthy adults (27 women and 12 men; age 23.7 ± 4.5) took part in the study. All had normal or corrected-to-normal vision and no history of neurological or psychiatric disorders. Five participants were excluded based on the same data quality criteria as in the original study: two completed fewer than 5 blocks; one contributed fewer than 200 trials after eye-tracking-based trial rejection; one showed poor overall behavioral performance (<50% accuracy); and one was left-handed. The final sample comprised 34 participants, which provided at least 80% power to detect effect sizes ranging from moderate to large (Cohen’s *f* > 0.25, using repeated-measures ANOVA). The study was approved by the local ethics committee (Faculty of Psychology and Educational Sciences, Ghent University). All participants provided informed consent before participation and received monetary compensation for their participation. Gender information was self-reported by participants. Data on socioeconomic status, communities of descent, and ethnicity were not collected.

#### Experimental apparatus

Stimuli were presented using the PsychoPy toolbox (Python 2.7) on a 24-inch LCD monitor (1280 × 1080 pixels; 60 Hz) at a viewing distance of 60 cm, with head position stabilized by a combined chin- and forehead-rest. The testing room was dimly lit and kept at a constant illumination to minimize visual distractions and maintain stable ambient lighting during eye tracking.

#### Experimental stimuli and paradigm

**Fig. 1:**
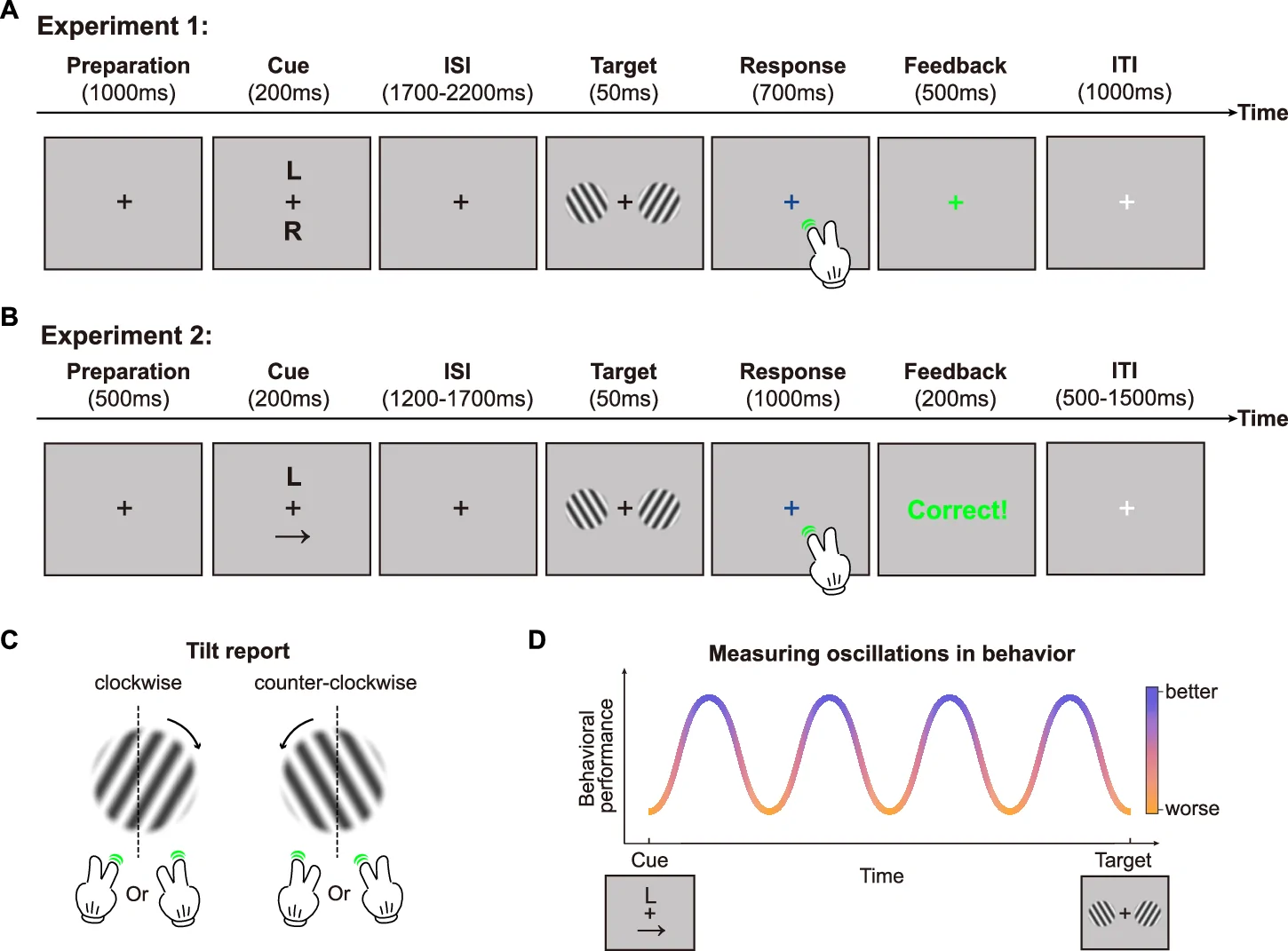
Paradigm. **A** Illustration of a single trial in Experiment 1. **B** Illustration of a single trial in Experiment 2. **C** Task display. Participants reported whether the target grating was tilted clockwise or counterclockwise relative to vertical, using the index or middle finger of the cued hand. **D** Behavioral performance (accuracy/reaction time) was sampled across cue–target intervals, yielding a time course on which rhythmic fluctuations can be assessed.

The task required participants to discriminate the orientation of a cued target grating (Fig. 1A). On each cue display, two letters were presented simultaneously with a size of 0.75° visual angle (vertical eccentricity of 1° visual angle): one above the fixation cross indicated which of the forthcoming gratings would be the target, and one below the fixation cross indicated which hand to use for the response. Cue letters were drawn from ‘L’ and ‘R’, yielding four combinations (LL, RR, LR, RL). LL and RR were defined as the easy (congruent) rule condition, whereas LR and RL constituted the difficult (incongruent) rule condition. On the subsequent target display, two sinusoidal gratings were presented (size of 5° visual angle; 10% contrast; 3 cycles per degree) on a mid-grey background, centered at 5° horizontal eccentricity to the left and right of fixation. The tilt angle was individually titrated using a staircase procedure (see below for details) to equate perceptual difficulty and avoid ceiling accuracy. Participants were requested to report the tilt of one of two gratings as being clockwise or counterclockwise from the vertical axis, using the index or middle finger of the instructed hand (Fig. 1C). On top of this, in order to assess rhythmic fluctuations in behavioral performance, we employed a dense-sampling design with evenly spaced inter-stimulus intervals (ISIs). ISIs ranged from 1700 to 2200 ms in 50 ms steps (11 levels) and were randomly assigned on each trial. Then, by aggregating accuracy and response time across ISIs (Fig. 1D), we were able to quantify oscillatory structure in behavior.

#### Single-trial sequence

Each trial began with a preparatory interval (1000 ms), followed by the cue display (200 ms). After a variable ISI, the target gratings appeared (50 ms). During the response window, the fixation cross changed from black to blue to indicate that participants should respond (maximum response time: 700 ms). Feedback was then provided for 500 ms via the fixation cross (green for correct, red for incorrect). Trials without a response were marked as misses and re-presented at the end of the block; the task was paused at the same time, and a prompt asked whether a break was needed. If no break was required, participants pressed the ‘space’ to proceed. An inter-trial interval (ITI) of 1000 ms then preceded the next trial.

#### Experimental procedure

Each participant first completed a practice block (80 trials; details are provided in Senoussi et al.^28^), followed by a staircase procedure (80 trials) to estimate an individual tilt level for use in the main task. Stimulus settings and timings were identical to the main task, with only grating orientation varied on a trial-to-trial basis, and trials were sampled across all cue types and ISIs to avoid ceiling performance. We used a one-up, two-down procedure with the tilt level initialized at 7° with 3° steps (ranging from 0.5 to 30°; minimum step 0.1°). The step size was halved at the first reversal and at every other reversal thereafter. Reversals happened at transitions from a run of correct responses to an error, or vice versa: after an error following correct responses, tilt increased (became easier); after two consecutive correct responses, tilt decreased (became harder). The tilt values carried into the main task were the mean of the final ten staircase values. The main task comprised 5–8 blocks, depending on participant pace (participants with fewer missed trials completed more blocks), corresponding to ~448–704 trials before trial rejection.

#### Data preprocessing and cleaning

The original dataset^28,31^ included behavioral, eye-tracking, and EEG recordings, but the present reanalysis focused only on the behavioral data. Eye-tracking data were used for trial rejection and were aligned with the behavioral logs using EEG triggers (acquisition and processing details are provided in Senoussi et al.^28^). In the current pipeline, we likewise rejected trials based on gaze position (epoched from cue onset to target onset) relative to the fixation cross, using a 1.5° threshold as in the original study. After data cleaning, trials were binned by condition (cue type) and by ISI. We then computed mean accuracy and mean RT within each bin, generating condition-specific time courses of behavioral performance across ISIs, that is, behavioral oscillations. Example single-participant time courses are shown in Supplementary Fig. 1.

#### Spectral analysis of behavioral oscillations

**Fig. 2:**
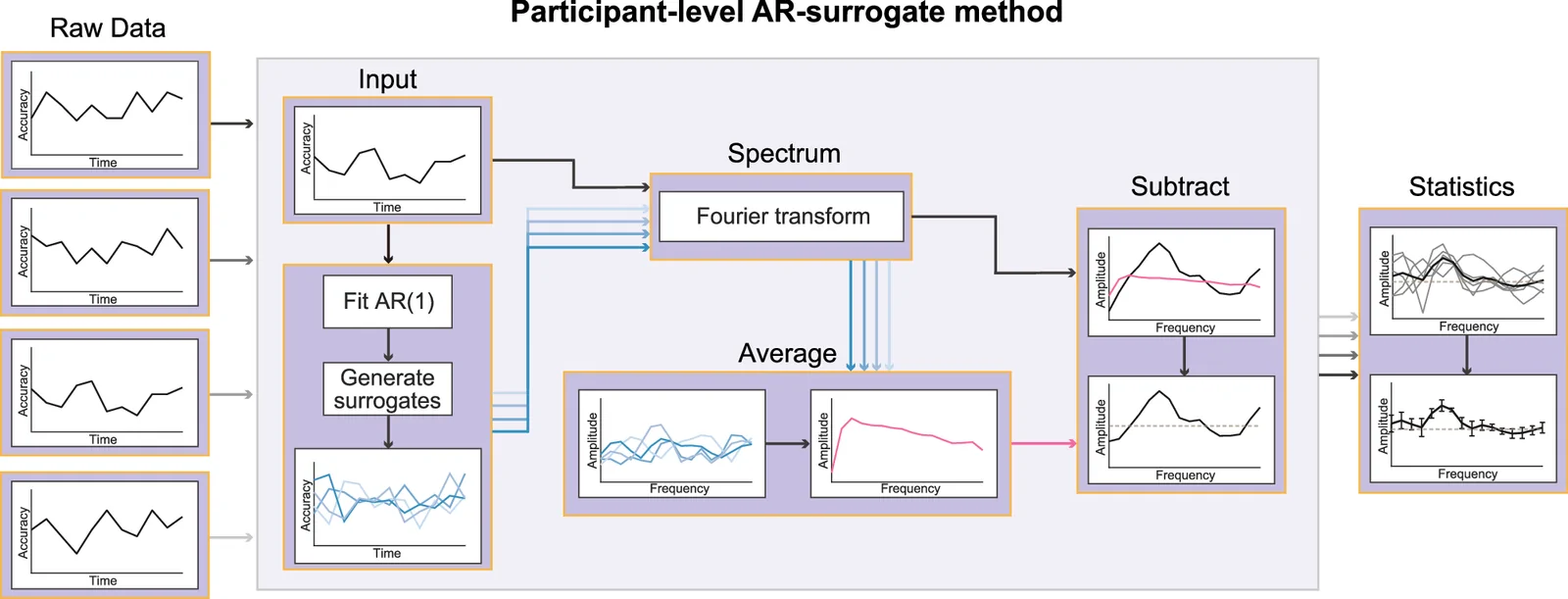
Analysis pipeline for participant-level AR-surrogate analyses. Participant-level behavioral time courses were constructed from accuracy or response time across cue–target intervals. An AR(1) model was fitted to each empirical time course to generate surrogate series capturing aperiodic temporal structure. Empirical and surrogate series were then submitted to Fourier analysis, and the average surrogate spectrum was subtracted from the empirical spectrum to obtain a deviation spectrum. Group-level statistics were applied to the deviation spectra to identify reliable rhythmic components. The figure is adapted from Harris and Beale^29^.

We used a state-of-the-art autoregressive approach (illustrated in Fig. 2)^29^ to estimate the spectral representation of behavioral oscillations. This method starts by fitting a first-order autoregressive (AR(1)) model to each participant’s ISI-binned time course^29^:

1 $${X}_{t}=\delta +\phi {X}_{t-1}+{\varepsilon }_{t}$$

We estimated the parameters (constant δ, autoregressive coefficient ϕ) per subject and simulated 1000 surrogate series of length *T* (with Gaussian noise ${\varepsilon }_{t}$) for each subject. These surrogates capture non-rhythmic trial-by-trial dependencies (e.g., priming). We then computed fast Fourier transform (FFT) amplitude spectra for the empirical series and all surrogates after zero-padding each time series with 32 samples (adding 16 zeros before and 16 after). For each frequency *f*, we defined the participant-level deviation spectrum as:

2 $$D(\,f)={A}_{{{{\rm{emp}}}}}(\,f)-{\mu }_{{{{\rm{surr}}}}}(\,f)$$

where ${A}_{{{{\rm{emp}}}}}(f)$ is the empirical amplitude and ${\mu }_{{{{\rm{surr}}}}}(f)$ is the mean amplitude across 1000 surrogates. Positive D(f) values indicate the presence of oscillatory activity.

Group-level rhythmicity was then assessed statistically on the participant-level deviation spectra, as detailed in the Statistical analysis section.

#### Statistical analysis

Statistical analyses were performed separately for accuracy and response time. To assess group-level rhythmicity, participant-level deviation spectra were tested against zero at each frequency bin using one-tailed one-sample *t* tests, because positive deviation values indicate stronger empirical spectral amplitude than expected from the participant-level AR-surrogate spectra. Benjamini–Hochberg false discovery rate (FDR) correction was applied across frequencies to control for multiple comparisons. For peak-frequency analyses, the theta peak was defined for each participant and condition as the frequency bin with the maximal deviation within the theta range. Peak-frequency estimates were compared across the four rule conditions using a 2 × 2 linear mixed-effects model with target location and response hand as fixed factors, and participants as random intercepts. We additionally compared easy rules (RR and LL) with difficult rules (LR and RL) to directly test the effect of rule difficulty. Participant-level directionality of the easy-versus-difficult difference was evaluated with a binomial test against chance level. Assumptions of the statistical analyses were evaluated using standard diagnostic procedures, including checks of normality, homogeneity of variance, and potential extreme observations.

### Experiment 2

#### Participants

Forty-two adults (32 women and 10 men; age = 25.83 ± 3.25) were recruited. All had normal or corrected-to-normal vision and no history of neurological or psychiatric disorders. Two participants were excluded (one for low accuracy and one for reversed response mapping), and fourteen were excluded based on predefined eye-movement criteria (see Data preprocessing and cleaning). The final sample comprised twenty-six participants, providing at least 80% power to detect experimental effect sizes ranging from moderate to large (Cohen’s *f* > 0.25, using repeated-measures ANOVA). The study was approved by the local ethics committee (Faculty of Psychology and Educational Sciences, Ghent University). All participants provided informed consent before participation and received monetary compensation for their participation. Gender information was self-reported by participants. Data on socioeconomic status, communities of descent, and ethnicity were not collected.

#### Experimental apparatus

Stimuli were presented with the PsychoPy toolbox (Python 3.10) on a 24-inch LCD monitor (1920 × 1080 pixels; 60 Hz) at a viewing distance of 93 cm, with head position stabilized by a combined chin and forehead rest. The testing room was dimly lit and kept at a constant illumination (~30 lux, measured with an illuminance meter) to minimize visual distractions and maintain stable ambient lighting during eye tracking. To ensure that task difficulty was not influenced by gaze position, participants were instructed to maintain fixation on a central fixation cross (size 0.30°). Eye movements of the dominant eye (monocular tracking) were recorded continuously with an EyeLink 1000 Plus desktop-mounted system (SR Research) at 1000 Hz, and all eye-tracking procedures were controlled via PsychoPy scripts. A 13-point calibration and validation procedure was performed at the beginning of each block, followed by a drift correction once per block (every 25 trials).

#### Experimental stimuli and paradigm

To ensure that any frequency shifts in behavioral oscillations reflected task difficulty rather than stimulus semantics, we modified the original design (see Experiment 1) to use letter–arrow cues instead of letters alone. Cue symbols were drawn from L, R, ←, →, yielding 16 two-symbol combinations. On each cue display, the instructions matched the original paradigm, with the upper symbol indicating the target location (left or right) and the lower symbol indicating the response hand. Cues were grouped by spatial congruency into three conditions: same-sym (same symbols: LL, RR, ←←, →→), same-side-dif-sym (same side, different symbols: L ← , ←L, R → , →R), and dif-sides (different sides: L → , LR, R ← , RL, ←R, ←→, →L, →←). To equate exposure across conditions, each participant viewed a counterbalanced subset of four dif-sides pairs (Fig. 1B). The two cue symbols were presented above and below fixation at ±0.75° vertical eccentricity, with a size of 0.75°. On the target display, a pair of sine-wave Gabor gratings was presented symmetrically at ±7.5° horizontal eccentricity (size 5°, contrast 0.2, spatial frequency 2 cycles per degree) on the mid-grey background. The task was identical to Experiment 1: participants reported whether the target grating was tilted clockwise or counterclockwise relative to vertical, using the index or middle finger of the instructed hand (Fig. 1C).

#### Single-trial sequence

Each trial began with a 500 ms preparation baseline, followed by presentation of the two cue symbols for 200 ms. A cue–target interval of 1200–1700 ms (step size 50 ms) then ensued. The target display was presented for 50 ms, and participants subsequently had 1000 ms to report whether the target grating was tilted clockwise or counterclockwise. Accuracy feedback was shown for 200 ms (green ‘Correct!’ for correct responses; red ‘Incorrect!’ for incorrect responses), after which an ITI of 500–1500 ms (step size 250 ms) preceded the next trial. Trials with no response were marked as misses and were re-presented at the end of the current block.

#### Experimental procedure

The experiment comprised two sessions on separate days. In each session, participants began with a brief ROI familiarization phase (~60 s) with gaze-contingent auditory feedback to encourage stable central fixation. During this phase, a central fixation marker (green dot; 20 × 20 pixels) was presented together with a circular ROI (size 1.5°), and a beep was triggered whenever the gaze deviated outside the ROI. In Session 1, participants then completed a practice block (100 trials) to familiarize themselves with the task (target duration 100 ms; response window 1500 ms), followed by a staircase block (100 trials) identical to that in Experiment 1. The resulting individual tilt angle from the staircase procedure was then used in the main task for both sessions. In Session 2, participants completed a shorter practice block (25 trials) and then proceeded directly to the main task. The main task across the two sessions comprised 1056 trials in total (428 in Session 1 and 628 in Session 2) and was organized into blocks of 25 trials each.

#### Data preprocessing and cleaning

We assessed fixation quality on every trial during the cue-to-target interval. A trial was marked as poor quality if gaze deviated by more than 2° from the central fixation cross at any time during this interval. Participants were excluded when more than 50% of their trials failed this criterion across the two sessions; in total, 14 participants were removed for low eye-tracking quality. At the session level, we required accuracy to be reliably above chance using a one-sided binomial test against 0.5 (α = 0.05). Two sessions (from two participants) did not pass this check and were excluded. The final dataset^32^ comprised all remaining sessions and included only valid trials (all miss trials excluded). Valid trials were then binned by condition (same-sym, same-side-dif-sym, and dif-sides) and ISI, and we computed mean accuracy and mean reaction time within each bin to prepare for the estimation of behavioral oscillations.

### Spectral analysis of behavioral oscillations

Identical to Experiment 1.

#### Subgroup analysis based on different-sides peak frequency

To examine the gap in the individual peak-frequency estimates in the dif-sides condition, we conducted a post hoc subgroup analysis. Participants were assigned to either the low- or high-frequency subgroup based on the median frequency bin in the accuracy-based dif-sides peak-frequency distribution. We then examined whether these subgroups differed in peak frequency, behavioral performance, and diffusion decision model (DDM) parameters, including boundary separation, drift rate, and non-decision time. DDM parameters were estimated using the EZ-diffusion method^33^ separately for each participant and condition, based on the proportion of correct responses, mean RT for correct trials, and RT variance for correct trials. Trials with RTs ≤0.2 s or ≥3 s were excluded before parameter estimation.

#### Statistical analysis

The deviation-spectrum and peak-frequency analyses followed the same procedures as in Experiment 1, except that peak-frequency estimates were compared across the three cue conditions (same-sym, same-side-dif-sym, and dif-sides). Behavioral performance was analysed with repeated-measures ANOVAs, followed by two-tailed paired *t* tests for pairwise condition comparisons. We also tested whether peak frequency predicted behavioral performance using generalized linear mixed models. For the subgroup analysis, peak frequency, behavioral performance, and DDM parameters were analysed using linear mixed-effects models with subgroup, condition, and their interaction as fixed effects, and participants as random intercepts. Follow-up comparisons between the low- and high-frequency subgroups within individual conditions were performed using Welch independent-samples *t* tests. Assumptions of the statistical analyses were evaluated using the same diagnostic procedures as in Experiment 1.

## Results

### Experiment 1

#### Replicating theta-rhythmic modulation of behavior

**Fig. 3:**
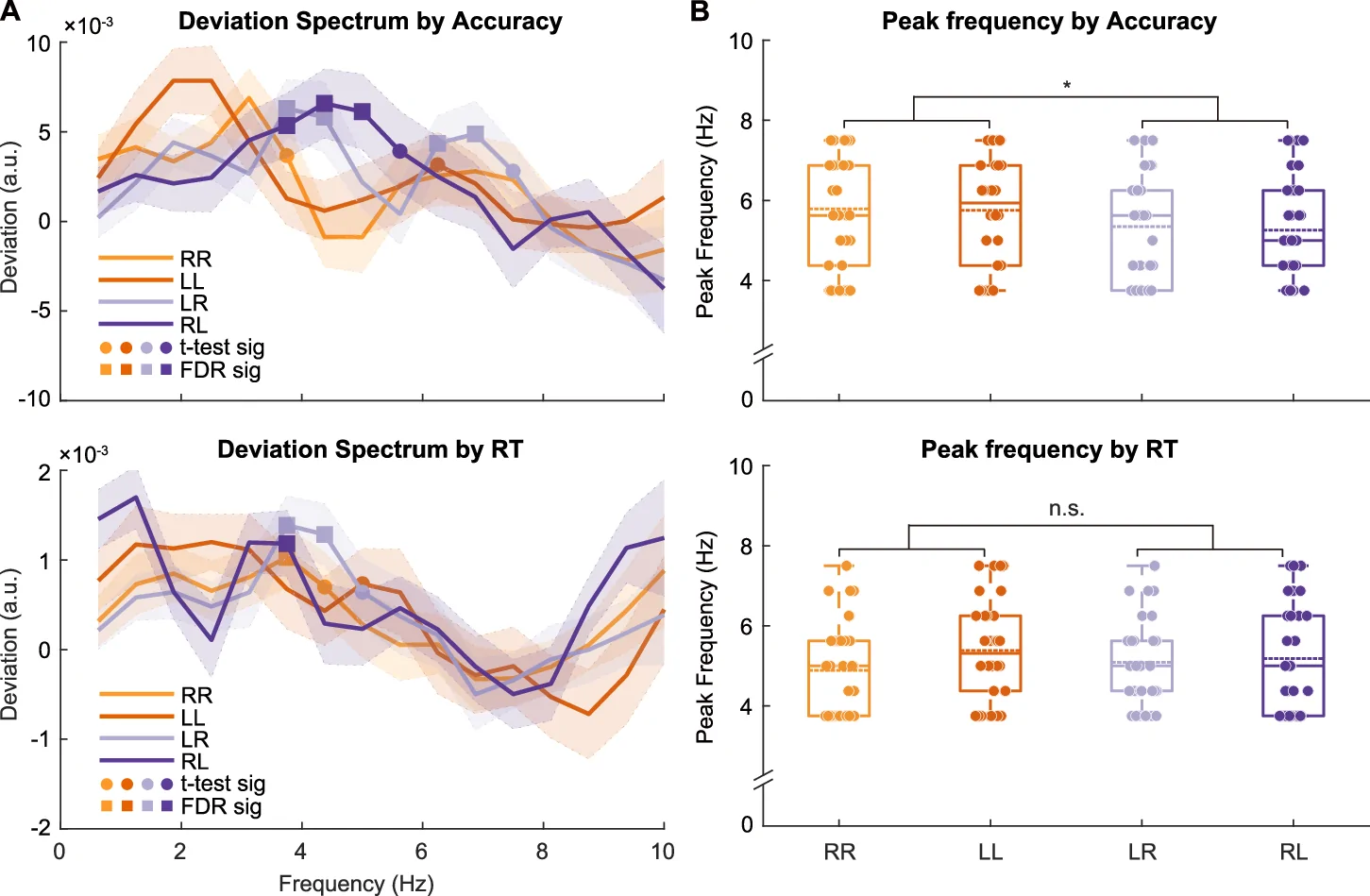
Results of Experiment 1 (*n* = 34 participants). **A** Group-mean deviation spectra for accuracy (top) and reaction time (bottom). Curves correspond to the four conditions (RR, LL, LR, RL); shaded areas indicate ± s.e.m. Filled circles mark frequencies significant by one-tailed one-sample *t* tests across participants (*P* < 0.05); filled squares mark frequencies surviving Benjamini–Hochberg FDR correction across frequencies (*q* = 0.05). Statistical analysis was restricted to the theta-frequency range (3.75–7.50 Hz). **B** Distributions of individual theta peak frequencies for accuracy (top) and reaction time (bottom). Box plots show the median and interquartile range (whiskers, 1.5 × IQR); points indicate individual participants; solid horizontal lines indicate the mean peak frequency. * indicates *P* < 0.05; n.s. indicates not significant.

All subsequent analyses were conducted on a robust dataset comprising 34 participants, following the exclusion of five individuals based on predefined data quality criteria (see “Methods”). Crucially, the oscillatory effects reported below were robust to these participant removal criteria. We began by verifying rhythmicity in the behavioral time courses of this published dataset^28,31^ by quantifying deviation spectra from participant-specific surrogate spectra (see Methods). Participant-level rhythmicity counts, defined by the presence of at least one positive theta-band deviation, are reported in Supplementary Table 1; full frequency-wise statistics within the analysed theta range are reported in Supplementary Table 2 for accuracy and Supplementary Table 3 for RT. In accuracy, clear oscillatory structure was evident across all four conditions (RR, LL, LR, RL; Fig. 3A, top), with somewhat stronger evidence under the difficult rules (LR and RL). Specifically, in the two easy rules (RR and LL) a single frequency bin reached *t* test significance but did not pass FDR correction (3.75 Hz in RR and 6.25 Hz in LL). By contrast, in LR five bins were statistically significant (3.75 Hz, 4.38 Hz, 6.25 Hz, 6.88 Hz, and 7.50 Hz), of which four remained significant after FDR correction (3.75 Hz: *t*_*(33)*_ = 3.89, *P* < 0.001, *p*_*FDR*_ = 0.002, Cohen’s *d*_*z*_ = 0.67, 95% CI [0.28, 1.05]; 4.38 Hz: *t*_*(33)*_ = 2.92, *P* = 0.003, *p*_*FDR*_ = 0.010, Cohen’s *d*_*z*_ = 0.50, 95% CI [0.13, 0.87]; 6.25 Hz: *t*_*(33)*_ = 2.81, *P* = 0.004, *p*_*FDR*_ = 0.010, Cohen’s *d*_*z*_ = 0.48, 95% CI [0.11, 0.85]; 6.88 Hz: *t*_*(33)*_ = 2.67, *P* = 0.006, *p*_*FDR*_ = 0.010, Cohen’s *d*_*z*_ = 0.46, 95% CI [0.09, 0.83]). Similarly in RL, *t* tests were significant in four bins, with three retaining significance after FDR correction (3.75 Hz: *t*_*(33)*_ = 2.96, *P* = 0.003, *p*_*FDR*_ = 0.007, Cohen’s *d*_*z*_ = 0.51, 95% CI [0.14, 0.88]; 4.38 Hz: *t*_*(33)*_ = 3.40, *P* < 0.001, *p*_*FDR*_ = 0.006, Cohen’s *d*_*z*_ = 0.58, 95% CI [0.20, 0.96]; 5.00 Hz: *t*_*(33)*_ = 2.92, *P* = 0.003, *p*_*FDR*_ = 0.007, Cohen’s *d*_*z*_ = 0.50, 95% CI [0.13, 0.87]). For RT, rhythmicity was likewise observed in all conditions (Fig. 3A, bottom): in RR, the *t* test was significant in two bins (3.75 Hz and 4.38 Hz), of which one bin was FDR-corrected significant (3.75 Hz: *t*_*(33)*_ = 3.01, *P* = 0.002, *p*_*FDR*_ = 0.017, Cohen’s *d*_*z*_ = 0.52, 95% CI [0.14, 0.89]); in LL, one bin reached uncorrected significance (5.00 Hz); in LR, three bins were significant (3.75 Hz, 4.38 Hz, and 5.00 Hz), of which two remained significant after FDR correction (3.75 Hz: *t*_*(33)*_ = 4.18, *P* < 0.001, *p*_*FDR*_ < 0.001, Cohen’s *d*_*z*_ = 0.72, 95% CI [0.32, 1.11]; 4.38 Hz: *t*_*(33)*_ = 3.74, *P* < 0.001, *p*_*FDR*_ = 0.001, Cohen’s *d*_*z*_ = 0.64, 95% CI [0.26, 1.03]); and in RL, one bin met the FDR-corrected threshold (3.75 Hz: *t*_*(33)*_ = 3.11, *P* = 0.002, *p*_*FDR*_ = 0.013, Cohen’s *d*_*z*_ = 0.53, 95% CI [0.16, 0.91]). Taken together, behavioral performance exhibits theta-band rhythmic fluctuation, which supports a true oscillatory process, possibly on top of aperiodic structure.

#### Reproducing the difficulty-dependent shift in peak frequency

**Fig. 4:**
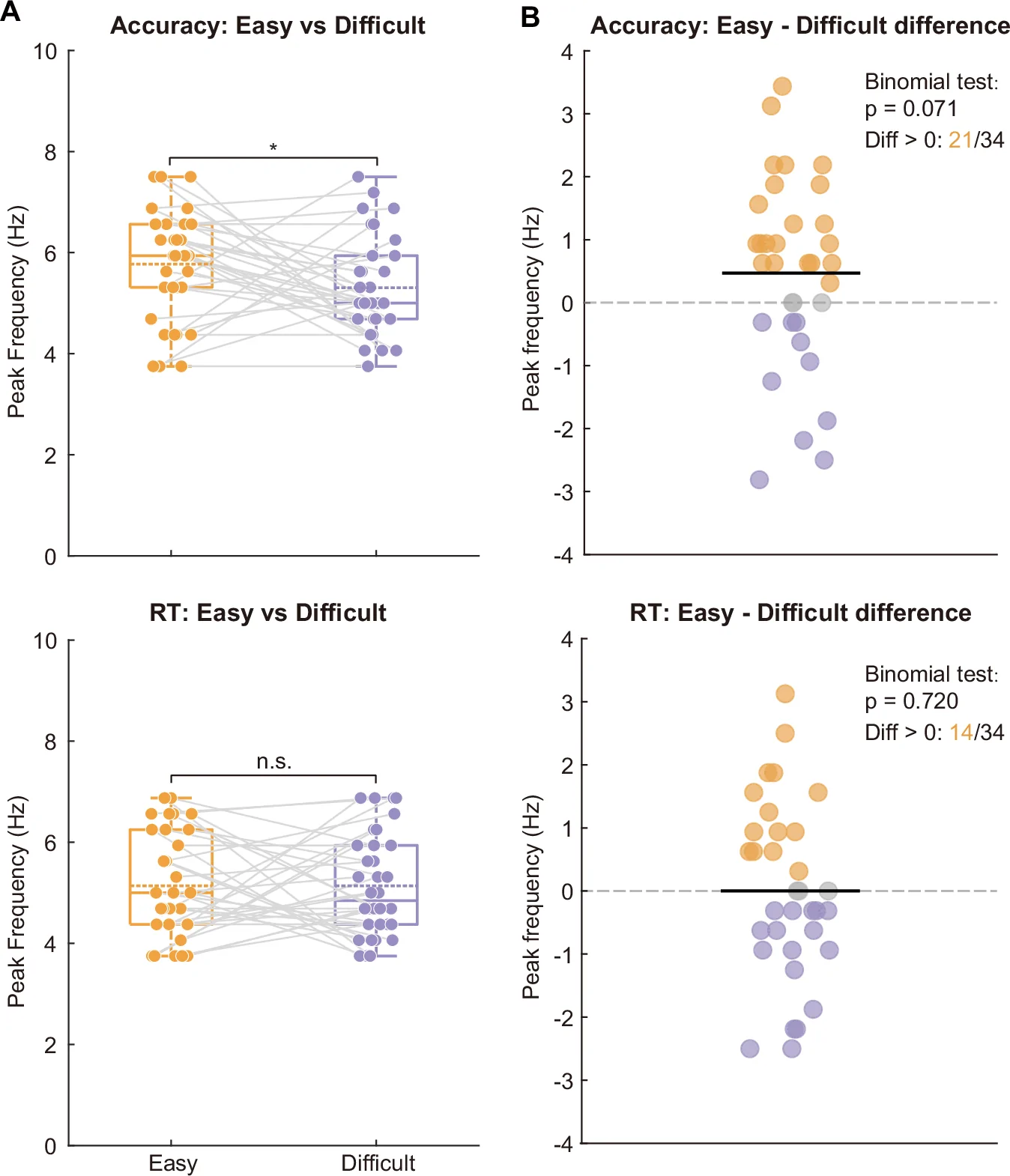
Peak frequency differences between easy and difficult rules (*n* = 34 participants). **A** Peak frequency for easy rules (RR and LL) and difficult rules (LR and RL), shown separately for accuracy-based estimates (top) and RT-based estimates (bottom). Dots represent individual participants, grey lines connect paired observations from the same participant, and box plots show the group distributions. The solid line within each box indicates the median, whereas the dotted horizontal line indicates the condition mean. **B** Participant-level differences in peak frequency (Diff = easy − difficult), shown separately for accuracy-based estimates (top) and RT-based estimates (bottom). Positive values indicate higher peak frequency for easy than difficult rules. The black horizontal line indicates the group mean difference, and the grey dashed line marks zero. Orange dots indicate participants with positive differences, purple dots indicate negative differences, and grey dots indicate zero differences. The inset reports the binomial test for the number of participants showing a positive difference. * indicates *P* < 0.05; n.s. indicates not significant.

A key observation in the earlier publication^28^ is the theta-frequency shift according to task demands, which we sought to replicate under the AR-model framework. Accordingly, we defined the peak frequency for each participant and condition as the frequency bin with maximal deviation and compared peak frequency across the four rule conditions using the 2 (hands) × 2 (target locations) linear mixed-effects model, separately for accuracy and RT (see “Methods” for details). Consistent with the original report, in accuracy, the 2 × 2 linear mixed-effects model showed a significant hand × target-location interaction (Type-III ANOVA with Satterthwaite df: *F*_*(1, 99)*_ = 4.25, *P* = 0.042, *η*_*p*_*²* = 0.04, 95% CI [0.00, 0.14]; Fig. 3B, top). To directly test the effect of rule difficulty, we additionally compared easy rules (RR and LL) with difficult rules (LR and RL). This follow-up linear mixed-effects model confirmed that peak theta frequency was lower for difficult rules than for easy rules (*F*_*(1, 101)*_ = 4.33, *P* = 0.040, *η*_*p*_*²* = 0.04, 95% CI [0.00, 0.14]; Fig. 4A, top). At the participant level, 21 of 34 participants showed a higher accuracy-based peak frequency for easy than difficult rules, indicating a marginal trend consistent with the group-level effect (binomial test, *P* = 0.071; Fig. 4B, top).

Parallel analyses using RT-based peak estimates did not show the same pattern. The 2 × 2 linear mixed-effects model did not provide significant evidence for a hand × target-location interaction (*F*_*(1, 99)*_ < 0.01, *P* = 1.000, *η*_*p*_*²* = 0.00, 95% CI [0.00, 0.00]; Fig. 3B, bottom). A Bayesian model comparison further provided moderate evidence against including this interaction (BF_01_ = 3.98). The corresponding easy-difficult comparison was also not significant (Fig. 4A, bottom), with only 14 of 34 participants showing the easy > difficult direction for RT-based peaks (binomial test, *P* = 0.720; Bayesian paired *t* test: BF_01_ = 5.44; Fig. 4B, bottom). Taken together, these results indicate that the frequency shift remains robust under the AR framework, which further supports that rule-dependent slowing of theta tracks cognitive-control demands at the behavioral level.

### Experiment 2

#### Behavioral performance

The final sample included 26 participants following predefined eye-tracking and binomial screening (see “Methods”). As part of this screening, participants were excluded when more than 50% of their trials were rejected based on the eye-tracking criteria. Across all screened participants, the eye-tracking-related trial exclusion rate was 39.30% ± 29.60% (mean ± SD; range = 2.42–97.94%). Among the included participants, this rate was 21.47% ± 16.42% (range = 2.42–49.91%; Supplementary Fig. 2). Of these, 24 participants contributed 1056 trials across both sessions, while 2 participants contributed 628 trials from Session 2 only. Critically, all reported behavioral effects remained robust to variations in participant exclusion criteria.

**Fig. 5:**
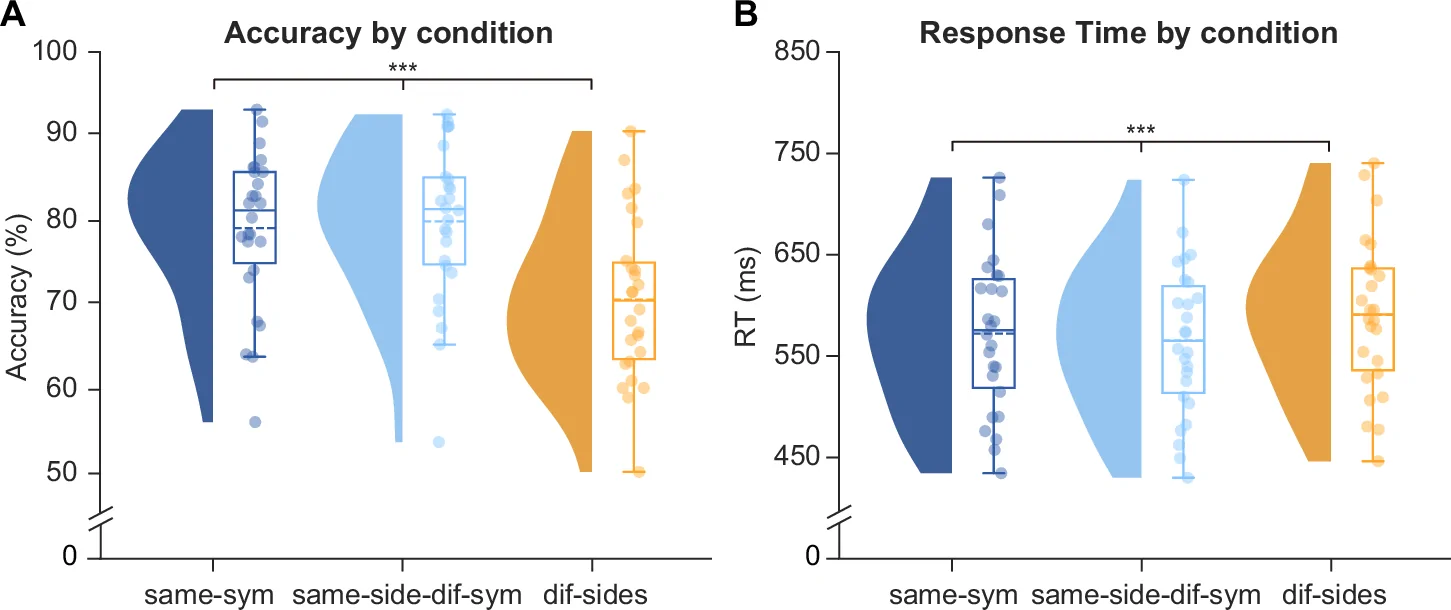
Group-level behavioral performance in Experiment 2 (*n* = 26 participants). **A** Accuracy across conditions. Violin plots show the across-participant distribution; box plots show the interquartile range (whiskers, 1.5 × IQR), with the solid line within each box indicating the median and the dashed horizontal line indicating the mean. Points indicate individual participants. **B** Similar to (**A**), but for response time. *** indicates *P* < 0.001.

Accuracy followed the expected prediction, same-side-dif-sym ≈ same-sym > dif-sides (means: same-sym = 79.16%, same-side-dif-sym = 79.94%, dif-sides =  70.69%; Fig. 5A). A repeated-measures ANOVA with Greenhouse–Geisser correction confirmed a condition effect (*F*_*(2,50)*_ = 15.88, *p*_*GG*_ < .001, *η*_*p*_*²* = 0.39, 95% CI [0.21, 0.59]). Two-tailed paired *t* tests showed lower accuracy for dif-sides relative to same-sym (*t*_*(25)*_ = 3.85, *P* < 0.001, Cohen’s *d*_*z*_ = 0.76, 95% CI [0.30, 1.22]) and same-side-dif-sym (*t*_*(25)*_ = 4.27, *P* < 0.001, Cohen’s *d*_*z*_ = 0.84, 95% CI [0.37, 1.31]), whereas same-sym and same-side-dif-sym did not differ (*t*_*(25)*_ = − 1.23, *P* = 0.229, Cohen’s *d*_*z*_ = −0.24, 95% CI [ − 0.65, 0.17]). RT showed a similar pattern, same-side-dif-sym < same-sym < dif-sides (means: same-side-dif-sym = 565.37 ms, same-sym = 572.24 ms, dif-sides = 590.83 ms; Fig. 5B). A repeated-measures ANOVA with Greenhouse–Geisser correction also showed a condition effect (*F*_*(2,50)*_ = 18.78, *p*_*GG*_ < 0.001, *η*_*p*_*²* = 0.43, 95% CI [0.23, 0.66]). Two-tailed paired *t* tests showed that same-side-dif-sym was faster than same-sym (*t*_*(25)*_ = 2.88, *P* = 0.008, Cohen’s *d*_z_ = 0.56, 95% CI [0.13, 1.00]) and that both same-sym and same-side-dif-sym were faster than dif-sides (same-sym vs dif-sides: *t*_*(25)*_ = − 3.37, *P* = 0.002, Cohen’s *d*_*z*_ = −0.66, 95% CI [ − 1.11, −0.21]; same-side-dif-sym vs dif-sides: *t*_*(25)*_ = − 5.79, *P* < 0.001, Cohen’s *d*_*z*_ = −1.14, 95% CI [ − 1.66, −0.61]). Together, the behavioral patterns show that dif-sides cues place the greatest control demands.

#### Replicating theta-rhythmic modulation of behavior

**Fig. 6:**
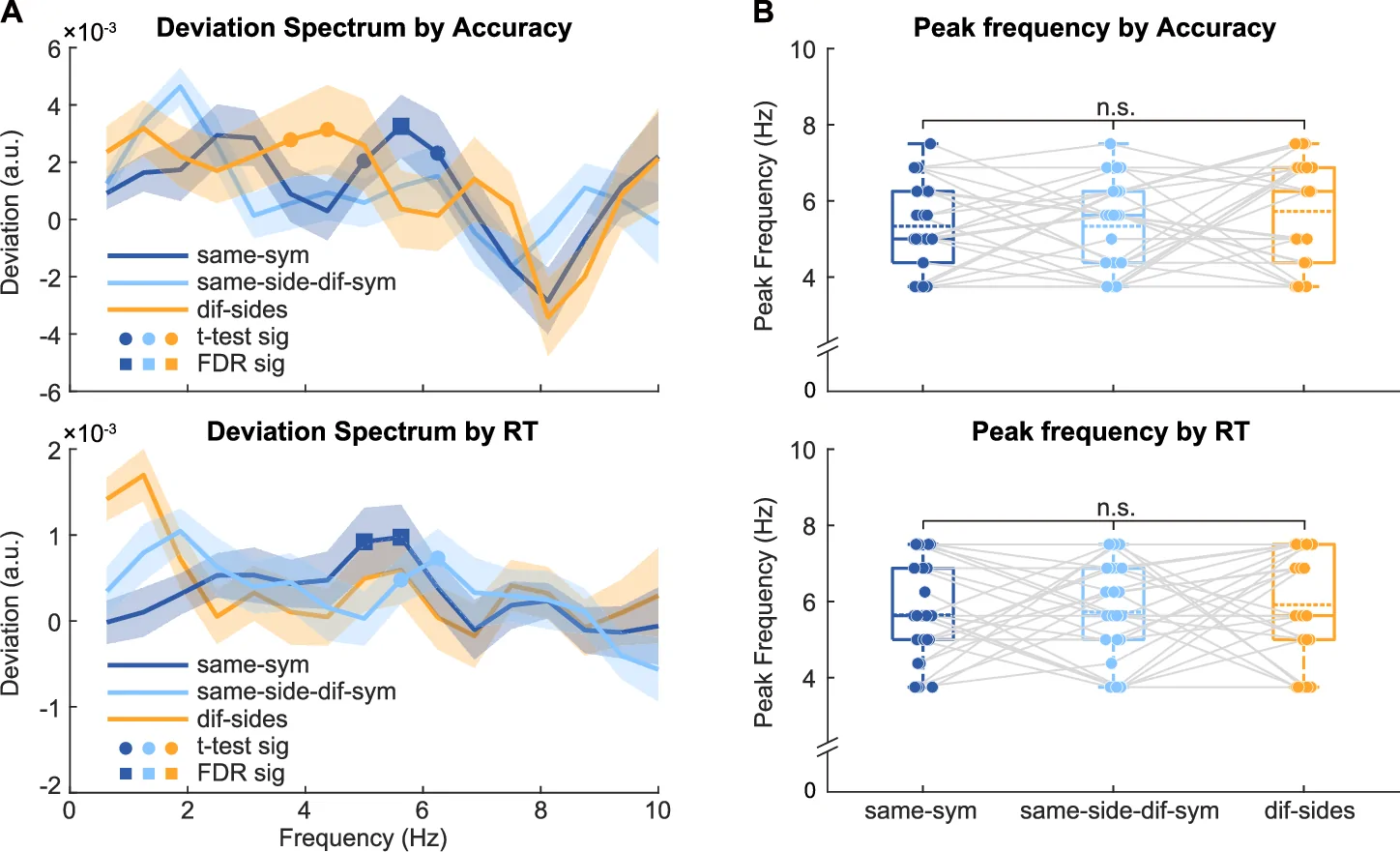
Results of Experiment 2 (*n* = 26 participants). **A** Group-mean deviation spectra for accuracy (top) and reaction time (bottom). Curves correspond to the three conditions (same-sym, same-side-dif-sym, and dif-sides); shaded areas indicate ±  s.e.m. Filled circles mark frequencies significant by one-tailed one-sample *t* tests across participants (*P* < 0.05); filled squares mark frequencies surviving Benjamini–Hochberg FDR correction across frequencies (*q* = 0.05). Statistical analysis was restricted to the theta-frequency range (3.75–7.50 Hz). **B** Distributions of individual theta peak frequencies for accuracy (top) and reaction time (bottom). Box plots show the interquartile range (whiskers, 1.5 × IQR), with the solid line within each box indicating the median. Points indicate individual participants, and dotted horizontal lines indicate the mean peak frequency. n.s. indicates not significant.

As in Experiment 1, we analyzed accuracy and RT by densely sampled cue–target intervals and, for each condition, computed deviation spectra relative to participant-level autoregressive surrogates to control aperiodic structure^29^. Participant-level rhythmicity counts for Experiment 2 are also reported in Supplementary Table 1, and full frequency-wise statistics within the analysed theta range are reported in Supplementary Table 4 for accuracy and Supplementary Table 5 for RT. Accuracy spectra exhibited theta-band rhythmic fluctuation (~4–8 Hz; Fig. 6A, top). In the same-sym condition, three significant frequency bins were observed: one at 5.62 Hz survived FDR correction (*t*_*(25)*_ = 2.96, *P* = 0.003, *p*_*FDR*_ = 0.023, Cohen’s *d*_*z*_ = 0.58, 95% CI [0.14, 1.02]), whereas the 5.00 Hz and 6.25 Hz bins were significant in the uncorrected *t* tests but did not survive FDR correction. In the dif-sides condition, two frequency bins reached *t* test significance (3.75 Hz and 4.38 Hz), but neither survived FDR correction. No frequency bin was significant in the same-side-dif-sym condition. RT spectra showed a similar pattern (Fig. 6A, bottom): two frequency bins were significant after FDR correction in same-sym (5.00 Hz: *t*_*(25)*_ = 2.33, *P* = 0.014, *p*_*FDR*_ = 0.049, Cohen’s *d*_*z*_ = 0.46, 95% CI [0.03, 0.88]; 5.62 Hz: *t*_*(25)*_ = 2.57, *P* = 0.008, *p*_*FDR*_ = 0.049, Cohen’s *d*_*z*_ = 0.50, 95% CI [0.07, 0.93]), whereas two frequency bins reached *t* test significance in same-side-dif-sym (5.62 Hz and 6.25 Hz) but did not survive FDR correction. Together, these analyses reveal a theta-band component in behavior, consistent with a common oscillatory mechanism in the same-sym condition.

#### No difficulty-dependent shift in peak frequency

In order to assess whether the reported theta-frequency shift also generalizes in this new dataset^32^, we applied the same analysis pipeline as Experiment 1 to extract, for each participant and condition, the theta peak defined as the frequency bin with maximal deviation in the accuracy and RT spectra. A linear mixed-effects model testing the main effect of condition did not provide significant evidence for differences in peak frequency across same-sym, same-side-dif-sym, and dif-sides for either accuracy (*F*_*(2,52)*_ = 0.94, *P* = 0.396, *η*_*p*_*²* = 0.04, 95% CI [0.00, 0.16]; Fig. 6B, top) or RT (*F*_*(2,78*)_ = 0.28, *P* = 0.753, *η*_*p*_*²* = 0.01, 95% CI [0.00, 0.06]; Fig. 6B, bottom). Bayesian repeated-measures analyses further supported this pattern, providing moderate evidence for the null model in both accuracy (BF_01_ = 4.22) and RT (BF_01_ = 7.17). We then examined whether peak frequency predicted performance using generalized linear mixed models with accuracy and RT as dependent variables to replicate another analysis from Senoussi et al.^28^. This analysis did not provide significant evidence that peak frequency predicted accuracy (*β* = −0.07, *z* = −1.40, *P* = 0.161, 95% CI [ − 0.17, 0.03]) or RT (*β* = 0.002, *z* = 0.20, *P* = 0.839, 95% CI [ − 0.02, 0.02]). Collectively, this new dataset^32^ does not provide clear evidence for systematic modulation of the behavioral theta peak. Thus, the results do not provide clear support for either a task-difficulty account or a cue ambiguity/entropy account.

#### Subgroup patterns based on different-sides peak frequency

**Fig. 7:**
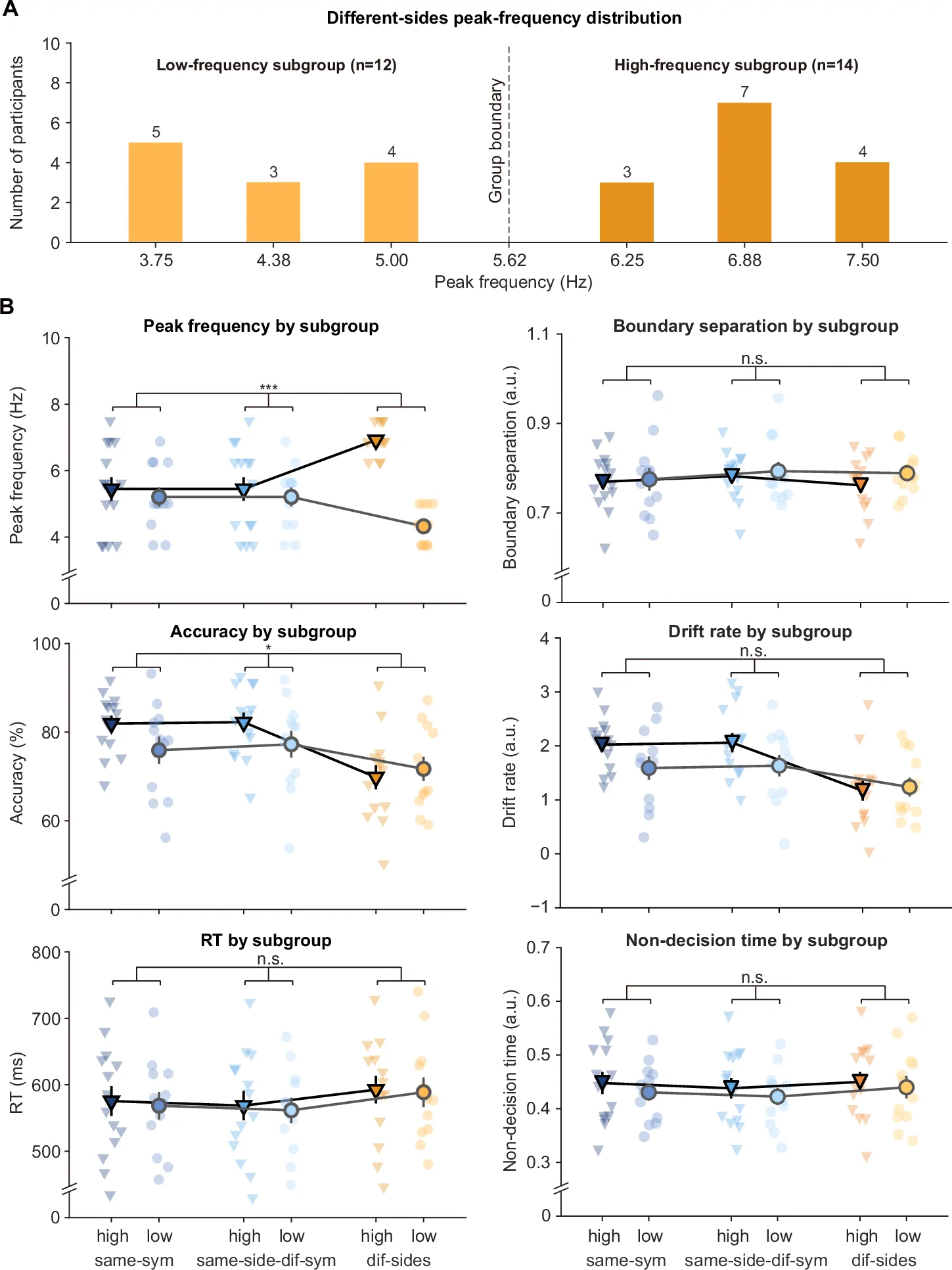
Peak-frequency, behavioral, and DDM measures in the low-frequency subgroup (*n* = 12 participants) and high-frequency subgroup (*n* = 14 participants). **A** Distribution of individual peak-frequency estimates in the different-sides condition. Participants were assigned to the low- or high-frequency subgroup according to whether their different-sides peak frequency fell below or above 5.62 Hz, marked by the dashed vertical line. Bars indicate the number of participants in each peak-frequency bin. **B** Peak-frequency, behavioral, and DDM measures for the low- and high-frequency subgroups across conditions. Panels show peak frequency (top left), boundary separation (top right), accuracy (middle left), drift rate (middle right), response time (RT; bottom left), and non-decision time (bottom right). Individual transparent points indicate participants. Triangles indicate the high-frequency subgroup, and circles indicate the low-frequency subgroup. Larger black triangles and grey circles connected by lines indicate subgroup means across conditions. *** indicates *P* < 0.001; * indicates *P* < 0.05; n.s. indicates not significant.

Because individual peak-frequency estimates in the dif-sides condition (Fig. 6B, top) appeared to cluster into low- and high-frequency ranges, we performed an exploratory analysis to examine whether this separation was associated with differences in behavior or DDM-derived parameters. Participants were assigned to low- and high-frequency subgroups according to whether their dif-sides peak frequency fell below or above the median value of 5.62 Hz (low-frequency subgroup: *n* = 12; high-frequency subgroup: *n* = 14; Fig. 7A), and subgroup effects were then tested across conditions using the linear mixed-effects models (see “Methods” for details).

As expected, peak frequency showed a significant subgroup effect (Type-III ANOVA with Satterthwaite df: *F*_*(1, 26)*_ = 19.49, *P* < 0.001, *η*_*p*_*²* = 0.43, 95% CI [0.14, 0.63]) and a significant subgroup × condition interaction (*F*_*(2, 52)*_ = 13.36, *P* < 0.001, *η*_*p*_*²* = 0.34, 95% CI [0.13, 0.50]), while the main effect of condition was not significant (*F*_*(2, 52)*_ = 0.83, *P* = 0.442, *η*_*p*_*²* = 0.03, 95% CI [0.00, 0.15]; Fig. 7B, top left). Follow-up Welch independent-samples *t* tests confirmed that the low- and high-frequency subgroups differed in the dif-sides condition (*t*_*(21.20)*_ = − 12.78, *P* < 0.001, Cohen’s *d* = −5.07, 95% CI [ − 6.76, −3.35]), but not in the same-sym (*t*_*(23.69)*_ = − 0.52, *P* = 0.609, Cohen’s *d* = −0.20, 95% CI [ − 0.96, 0.57]) or same-side-dif-sym conditions (*t*_*(23.48)*_ = − 0.53, *P* = 0.604, Cohen’s *d* = −0.20, 95% CI [ − 0.97, 0.56]).

We then asked whether this subgroup split was reflected in behavioral performance. For accuracy, the linear mixed-effects model showed a significant condition effect (*F*_*(2, 52)*_ = 17.32, *P* < 0.001, *η*_*p*_*²* = 0.40, 95% CI [0.19, 0.56]) and a subgroup × condition interaction (*F*_*(2, 52)*_ = 3.25, *P* = 0.047, *η*_*p*_*²* = 0.11, 95% CI [0.00, 0.27]), but no main effect of subgroup (*F*_*(1, 26)*_ = 1.08, *P* = 0.309, *η*_*p*_*²* = 0.04, 95% CI [0.00, 0.26]; Fig. 7B, middle left). This pattern suggests that the high-frequency subgroup showed a stronger accuracy decrease in the dif-sides condition, although the two subgroups did not differ significantly within any single condition (same-sym: *t*_*(17.97)*_ = − 1.68, *P* = 0.111, Cohen’s *d* = −0.67, 95% CI [ − 1.48, 0.15]; same-side-dif-sym: *t*_*(20.52)*_ = − 1.34, *P* = 0.194, Cohen’s *d* = −0.53, 95% CI [ − 1.32, 0.27]; dif-sides: *t*_*(23.84)*_ = 0.49, *P* = 0.626, Cohen’s *d* = 0.19, 95% CI [ − 0.58, 0.96]). RT showed only a condition effect (*F*_*(2, 52)*_ = 19.69, *P* < 0.001, *η*_*p*_*²* = 0.43, 95% CI [0.22, 0.58]), with no subgroup effect (*F*_*(1, 26)*_ = 0.04, *P* = 0.838, *η*_*p*_*²* = 0.00, 95% CI [0.00, 0.13]) or subgroup × condition interaction (*F*_*(2, 52)*_ = 0.10, *P* = 0.908, *η*_*p*_*²* = 0.00, 95% CI [0.00, 0.05]; Fig. 7B, bottom left).

For the DDM parameters, boundary separation did not show significant effects of subgroup (*F*_*(1, 26)*_ = 0.42, *P* = 0.521, *η*_*p*_*²* = 0.02, 95% CI [0.00, 0.21]), condition (*F*_*(2, 52)*_ = 1.50, *P* = 0.232, *η*_*p*_*²* = 0.05, 95% CI [0.00, 0.19]), or their interaction (*F*_*(2, 52)*_ = 0.71, *P* = 0.498, *η*_*p*_*²* = 0.03, 95% CI [0.00, 0.14]; Fig. 7B, top right). Drift rate showed a significant condition effect (*F*_*(2, 52)*_ = 19.66, *P* < 0.001, *η*_*p*_*²* = 0.43, 95% CI [0.22, 0.58]), and visually followed a similar pattern to accuracy, although neither the subgroup effect (*F*_*(1, 26)*_ = 1.64, *P* = 0.212, *η*_*p*_*²* = 0.06, 95% CI [0.00, 0.29]) nor the subgroup × condition interaction reached significance (*F*_*(2, 52)*_ = 3.12, *P* = 0.052, *η*_*p*_*²* = 0.11, 95% CI [0.00, 0.27]; Fig. 7B, middle right). Non-decision time showed a condition effect (*F*_*(2, 52)*_ = 4.96, *P* = 0.011, *η*_*p*_*²* = 0.16, 95% CI [0.01, 0.33]), but no subgroup effect (*F*_*(1, 26)*_ = 0.33, *P* = 0.572, *η*_*p*_*²* = 0.01, 95% CI [0.00, 0.20]) or subgroup × condition interaction (*F*_*(2, 52)*_ = 0.33, *P* = 0.717, *η*_*p*_*²* = 0.01, 95% CI [0.00, 0.10]; Fig. 7B, bottom right).

Overall, the subgroup split was most clearly reflected in the dif-sides peak-frequency estimates themselves. Beyond this defining difference, there was limited evidence for systematic subgroup differences in behavior or DDM parameters, although the high-frequency subgroup showed a stronger dif-sides-related decrease in accuracy.

## Discussion

The present study revisited accounts of rhythmic cognition by using a state-of-the-art autoregressive (AR) surrogate approach^29^ on two behavioral datasets. In Experiment 1 (Fig. 1A), a reanalysis of a previously published dataset^28,31^ revealed robust theta-band oscillations in both accuracy and response time (Fig. 3A), even after explicitly modelling and removing each participant’s aperiodic temporal structure. Using this more conservative pipeline, we replicated the earlier finding^28^ that the peak theta frequency of accuracy slows under more demanding rule conditions (Fig. 3B, top). In Experiment 2, we used a modified cue design that separated cue ambiguity from task difficulty (Fig. 1B). This dataset^32^ also showed evidence for theta-band rhythmicity in behavioral performance, although the pattern was weaker than in Experiment 1 (Fig. 6A). Moreover, unlike Experiment 1, Experiment 2 showed no reliable frequency shifts as task demands changed (Fig. 6B). Taken together, these results strengthen the evidence that behavioral time courses contain genuine theta-band oscillations, but find no additional evidence that the frequency of behavioral rhythms shifts with task difficulty.

These results raise a broader mechanistic question: how can oscillations help keep concurrent streams of information apart during cognition? Many theories converge on the idea that a core function of oscillations is to minimize interference between representations (e.g., “Communication through Coherence”)^1,34^. In such accounts, oscillatory dynamics organize information processing into recurring temporal windows, with distinct phases within a single cycle assigned to different representations^25,35,36^. This temporal segregation prevents overlap between competing representations^37,38^, while still supporting effective feature binding^39,40^. Functionally, this temporal organization may help coordinate sensory selection and response preparation^5,6^, allowing selected sensory input to be linked to the appropriate response while competing mappings are kept apart. In our paradigm, participants had to select one of two sensory inputs and map it onto a left- or right-hand response. Because all information will be partially activated across trials, temporal binding of the currently relevant task processes (e.g., for cue LR, left stimulus and right hand) via rhythmic alternation can help reduce representational interference and help to address the binding problem^41,42^. Our observation of behavioral theta-frequency fluctuations in Experiment 1, together with the persistence of rhythmicity in Experiment 2 even without a reliable frequency shift, supports the notion that such phase-based segregation is a fundamental strategy for minimizing interference in cognition.

### Limitations

The successful recovery of the previously reported frequency shift in Experiment 1 helps frame the null frequency-shift result in Experiment 2. It suggests that the absence of a reliable shift in Experiment 2 should not be attributed to the analysis approach itself. Instead, the discrepancy between the two experiments points to possible limits in estimating behavioral frequency modulation. Indeed, whereas neural recordings provide substantial evidence for rhythmicity and frequency modulation^17,18,43–47^, their behavioral signatures are harder to estimate reliably. In Experiment 2, theta-band rhythmicity was present, but it was less consistently expressed across conditions and measures than in Experiment 1. One reason may be that the revised cue design, while useful for separating cue ambiguity from task difficulty, also required each condition type to be pooled across several cue combinations. This may have made the behavioral time courses more heterogeneous than in the original paradigm. Moreover, although dense-sampling designs are powerful for detecting rhythmicity^26,48,49^, the number of observations per participant remains limited once trials are divided by condition and interval. Thus, the weaker rhythmicity in some Experiment 2 conditions should not be interpreted as evidence that behavioral theta rhythms were absent in those conditions. Rather, it may reflect greater variability in condition-level time courses arising from both the pooling across cue combinations and the limited number of observations per interval.

This issue also carries over to the frequency-shift analysis. Detecting a shift in peak frequency requires stable rhythmic estimates within each condition before those estimates can be compared across task demands. If rhythmicity is weaker or more variable in some conditions, the extracted peak frequencies will also be less precise. Therefore, the null result of the frequency-shift analysis in Experiment 2 does not rule out frequency modulation; it may instead reflect insufficient within-participant data or greater within-condition variability, making it difficult to estimate condition-specific peaks with confidence. In addition, the AR(1) surrogate model is itself a relatively simplified assumption about behavioral temporal dynamics^29,30^. Real behavioral time courses may contain higher-order or nonlinear dependencies that are not captured by this model, which could in turn bias the surrogate-derived baseline^50^ and thereby affect estimates of true periodicity.

These limitations point to clear directions for methodological improvement. One straightforward step is to adopt multi-session designs^51^, in which participants complete many more trials spread across multiple sessions. Such designs (with more data points per subject) would allow more precise estimation of individual oscillatory profiles (e.g., peak frequency^52^) and could also reduce the impact of between-subject variability. In parallel, efforts that combine data from multiple laboratories or multiple datasets through meta-analysis^53^ could help clarify whether frequency shifts reliably appear under specific task demands^18,54^, sampling schemes^55^, or analysis pipelines (to separate periodic and aperiodic components)^29,30,56^. A meta-analytic approach would also allow formal quantification of effect sizes and heterogeneity across paradigms, which is difficult to assess from single studies^57,58^.

In summary, applying rigorous and robust (AR-based) state-of-the-art analyses to both a published^28,31^ and a new dense-sampling dataset^32^ provides converging evidence that cognition is intrinsically rhythmic, although adaptive modulation of such rhythms remains to be investigated using different tasks, difficulty manipulations, and more powerful statistical and analytical approaches. These findings suggest that the brain organizes behavior not as a continuous flow, but as a series of discrete, rhythmically gated operations that support efficient processing by minimizing interference in cognition.

## Supplementary information

Transparent Peer Review file

Supplementary Information

## Data Availability

All data and research materials for Experiment 1, originally associated with Senoussi et al.^28^, are available at https://osf.io/nwh87/?view_only=b11ee1f860804da582c816fe8acdecad. All data and research materials for Experiment 2 are available at https://osf.io/3k2r5/?view_only=949efc2399f04e079d8964037243692d.

## Code availability

All code used to run the experiments and perform the analyses for Experiments 1 and 2 is available at https://doi.org/10.5281/zenodo.20053285.
