# Supplementary material for: Robust evidence for theta-band rhythmicity in behavior across two dense-sampling datasets
Source: Commun Psychol. 2026 Jul 7;4:155. doi: 10.1038/s44271-026-00500-0 (PMC13728126; doi:10.1038/s44271-026-00500-0)
Supplement: Supplementary file 1 [file 44271_2026_500_MOESM1_ESM.pdf]

nature portfolio

Peer Review File

Robust Evidence for Theta-Band Rhythmicity in Behavior across Two Dense-Sampling Datasets

Corresponding Author: Ms Mengting Xu

This file contains all editorial decision letters in order by version, followed by all author rebuttals in order by version.

Version 0:

Decision Letter:

** Please ensure you delete the link to your author homepage in this e-mail if you wish to forward it to your coauthors **

Dear Ms Xu,

Thank you for your patience during the peer-review process. Your manuscript titled "Rhythmicity Revisited: Evidence from Robust Behavioral Oscillations" has now been seen by 3 reviewers, and I include their comments at the end of this message. They find your work of interest but raised some important points. We are interested in the possibility of publishing your study in Communications Psychology, but would like to consider your responses to these concerns and assess a revised manuscript before we make a final decision on publication.

We therefore invite you to revise and resubmit your manuscript, along with a point-by-point response to the reviewers. Please highlight all changes in the manuscript text file.

Editorially, we consider it important that the authors address all reviewer concerns, in particular the need to better motivate the reanalysis and attempted replication, clarify the added value of the findings, and include additional analyses and controls to address differences across datasets and potential individual variability.

Please ensure you follow our statistical guidelines when reporting statistics (https://www.nature.com/commspsychol/submit/submission-guidelines#statistical-guidelines). Please note in particular our requirements for the reporting and interpretation of null-results. Non-significant findings derived from null-hypotheses significance tests should be reported in full, but may not be interpreted. Where you interpret null results, this interpretation must be based on Bayes Factors or equivalence tests.

As you revise the manuscript in response to these issues, please also implement all requests in the attached Mandatory Revision Requests document. All requirements listed in this document need to be fully met, or the work will be returned to you for further revisions without peer review. This workflow is in place to increase the likelihood that the paper will be accepted for publication. It reduces the number of rounds of revision (and review) and ensures that the reviewers vet a version of the article that is compliant with journal policies. If you have any questions regarding the required revisions, please contact the journal prior to resubmission to avoid a negative outcome.

We are committed to providing a fair and constructive peer-review process. Please don't hesitate to contact us if you wish to discuss the revision in more detail.

Please submit the following items:

- Revised manuscript
- Point-by-point response to the referees’ comments
- Mandatory Revision Requests Table (attached).
- Cover letter (as a separate document)

via this link: Link Redacted .

** This url links to your confidential home page and associated information about manuscripts you may have submitted or are reviewing for us. If you wish to forward this email to co-authors, please delete the link to your homepage first **

We hope to receive your revised paper within 8 weeks; please let us know if you aren't able to submit it within this time so that we can discuss how best to proceed. If we don't hear from you, and the revision process takes significantly longer, we may close your file. In this event, we will still be happy to reconsider your paper at a later date, provided it still presents a significant contribution to the literature at that stage.

We look forward to seeing the revised manuscript and thank you for the opportunity to review your work.

Best regards,

Troby Lui, on behalf of

David Pascucci

Troby Lui, PhD
Associate Editor
Communications Psychology

David Pascucci
Guest Editor
Communications Psychology

REVIEWER EXPERTISE:
Reviewer #1: Behavioural rhythms, brain oscillations, dense sampling and rhythmic analysis
Reviewer #2: Behavioural rhythms, brain oscillations, dense sampling and rhythmic analysis
Reviewer #3: Behavioural rhythms, brain oscillations, dense sampling and rhythmic analysis

REVIEWER REPORTS:

Reviewer #1 (Remarks to the Author):

The paper title "Rhythmicity Revisited: Evidence from Robust Behavioral Oscillations" reports the application of contemporary methodologies for detecting behavioral oscillation to a previously published dataset as well as an empirical attempted replication. I commend the authors for this project and find it is the type of work that generally help the field move forward, responsibly.
The background task and reference datasets are clarified well. The methodology generally clear. And the findings support the conclusions. Limitations of the study are also discussed well including marking horizons for future directions within the quest to fully validate past findings.
1. Although the paper in essence is a reanalysis and attempted replication – I think that the authors can do a bit better in fully motivating the study. Generally, the introduction and the discussion prioritize linking the work to neuronal oscillations touching on a broad gamut of whether those are or aren't relevant. I wonder whether the literature on cognitive models of sensory processing and response selection might make for a more natural background for the work. Of course considering the behavioral rhythms, those could link to models on how and why such cognitive functions might engage in fluctuations (Fiebelkorn et al., 2019 as well as Re et al., 2025 provide nice theoretical frameworks for those).
2. The title of the paper seems to promise a bit more than what thie report actually is. Rhythmicitiy isn't quite revisited here. The paper provides a validation of previous findings rather than challenge. Re-evaluation if anything in the most literal sense rather than a revisitation that sound more provocative than what this actually delivers.
3. Including a diagram of the analysis pipeline would be lovely. Indeed, the paper referenced has such a diagram, however, including a similar diagram here would make a good educational point – which would add value to this publication
4. inclusion of time series data. If possible it would be really nice to see examples of accuracy and RT time courses. I realize that averaging those for the group might be tricky considering the approach (and perhaps the nature of the data). But having an example subject would be a nice addition for the readers to gain intuitive sense of what the spectra consist of.
5. Forteen participants were removed due to more than 50% trial exclusion in experiment 2 (due to eye movements). What are the stats of eye movements in the included participants?
6. The diagram for tilt report is either confusing or erroneous. It looks like its labeled opposite than what I would deem CW or CCW tile off vertical
7. Might the authors consider indicating significance to the peak frequency barplot as well?

Reviewer #2 (Remarks to the Author):

The manuscript "Rhythmicity revisited: Evidence from Robust Behavioral Oscillations" by Xu et al. presents an analysis of two datasets to investigate the presence of rhythmicity in behavioral data. The authors use a previously published method [Harris & Beale, 2025] to re-analyze dataset #1 (previously published, Senoussi et al., 2022) and a new unpublished dataset #2. In dataset #1, the authors confirm the previously published results by finding evidence for rhythmicity in the theta range (4-8 Hz) as well as evidence for shifts in frequency across conditions. In dataset #2, the authors state that they find robust evidence for rhythmicity in the theta range, but it's only present in one of the three experimental conditions after multiple comparison correction. No evidence for task-related frequency shifts of behavioral rhythms.

I find the research question very interesting and the aim to confirm previous results great from an open and robust science perspective. Code and data availability is also a great aspect of the manuscript. I do think more work is needed to clarify methodological aspects and inconsistent results in the manuscript, as well as potential implications of the results.

General:

The reanalysis of the dataset #1 confirms the previous results. But do we learn more from the improved analysis? Does it have any added value? At the moment, the authors present group-level results, but as I understand the method, also individual level statements can be made, e.g. how many participants show significant behavioral rhythmicity, are there shifts on the individual level, etc. Given the usual interindividual variability in such data, I think it would be great to include also more analysis on the individual level.

The behavioral rhythms do not seem to be very consistent, as dataset #2 seems to indicate that behavioral rhythms cannot be detected reliably across all conditions. It's also tricky to say because the conditions are not compared directly. But the reader is left wondering if and why this is the case, as the discussion simply states that the authors "again observed theta-band rhythmicity in behavioral performance" for experiment #2, without further discussion of the potential differences between the two datasets. This does not seem like a sufficient analysis and discussion of the results.

Other:

- Fig 2: It would be great to illustrate the procedure of the method in more detail. e.g. showing the process + individual level data in Fig. 2. This could make the method more accessible to readers who are not familiar with it, without having to consult Harris & Beale (2025) for details, and could also illustrate data quality.
- The analysis was done for RT & accuracy in conjunction. Are there different expectations in terms of rhythmicity for these two measures? E.g. Fig. 2A shows quite an increase at about 10 Hz for the deviation spectrum for RT, that is cropped out in the analysis, as the "statistical analysis was restricted to theta-frequency range (3.75 - 7.50 Hz)" (also this seems to be a deviation from the theta-band range stated in the introduction; why?).
- Fig 2B/4B: why is the frequency z-scored? Maybe it would be more informative to show the actual frequencies. Also possibly show the paired relationship between the conditions.
- Fig 3: maybe plot tests performed + outcomes for the significant difference in the figure for a better overview of the results.
- labels: SS, SSiDS, DSi: maybe more verbose labels for figures or different more verbose naming of conditions would make the article easier to read. Also, abbreviations like CCW, CW & others do not appear often, consider writing them out for legibility.
- It is not clear how much data was available in dataset #1, e.g. the number of trials is not mentioned. When I checked the original Senoussi et al. paper, I saw also that EEG data was collected. It would be good to mention, even if not analyzed here, to give a better picture of the structure of the dataset.

Reviewer #3 (Remarks to the Author):

In this work, Xu and colleagues set out to assess two questions: 1) whether behavioural timecourses contain true theta-band oscillations, and 2) whether theta-rhythmic behavioural oscillations slow their peak frequency as task difficulty increases, or whether such slowing, observed in a previous study, might be due to effects such as cue ambiguity. They performed a reanalysis of old data (labelled Experiment 1) using a new analysis method and confirmed the theta rhythmicity of behaviour and the slowing of peak frequency in the difficult task condition (responding with the incongruent hand relative to the response direction). Experiment 2 replicated the theta-frequency rhythmicity in behaviour but produced no differences in peak frequency between the easy low ambiguity condition (SS), the easy high ambiguity condition (SSiDS), and the hard high ambiguity condition (DSi). The authors conclude from this that theta-rhythmic behavioural oscillations are a robust phenomenon, but that their modulation by task difficulty may not be.

This is a very well-written manuscript that assesses an interesting and timely question with appropriate methods. I make a couple of suggestions for ways I think the paper could be improved below.

Major:

- On my first readthrough, I misinterpreted the authors' conclusions regarding Experiment 2 as being in support of the cue ambiguity/entropy account. This was because they set the experiment up as testing competing difficulty versus ambiguity/entropy hypotheses, and when there were no differences, they concluded there were no difficulty effects. As the ambiguity hypothesis was not discussed any further, I took this at first to be the implicit winning hypothesis. I think the manuscript would be clearer if the authors also discussed the ambiguity/entropy account and stated for the reader's benefit that this account was also not supported.

- In figure 4B, the data for the DSi condition looks quite bimodal for both measures. It might be fruitful to perform an exploratory analysis where you split the data between the high and low peak-frequency clusters and look at whether there might be subsets in the data that are showing different patterns.

Minor:

- Lines 122-123: "inter-trial intervals" should be "inter-stimulus intervals"

- Throughout the Results, cases with multiple significant frequencies are described as multiple "significant peaks" when, from the plots, they appear in each case to be multiple points spanning a single peak. Perhaps this could be reworded?

- Line 45: reference 13 (Fiebelkorn et al., 2013, Current Biology) is a behavioural oscillations study. As they did not measure brain activity, their conclusions cannot support the relationship between instantaneous phase and perception/attention.

- Lines 44-45: Instead of the above, may I shamelessly suggest a citation of my own work in this section:

o Attention & perception: Harris, A. M., Dux, P. E., & Mattingley, J. B. (2018). Detecting unattended stimuli depends on the phase of prestimulus neural oscillations. Journal of Neuroscience, 38(12), 3092-3101.

o Perception: Williams, J. G., Harrison, W. J., Beale, H. A., Mattingley, J. B., & Harris, A. M. (2024). Effects of neural oscillation power and phase on discrimination performance in a visual tilt illusion. Current Biology, 34(8), 1801-1809.

Signed,
Anthony M. Harris

* TRANSPARENT PEER REVIEW: Communications Psychology uses a transparent peer review system. This means that we publish the editorial decision letters including Reviewers' comments to the authors and the author rebuttal letters online as a supplementary peer review file. However, on author request, confidential information and data can be removed from the published reviewer reports and rebuttal letters prior to publication. If your manuscript has been previously reviewed at another journal, those Reviewers' comments would not form part of the published peer review file.

** Visit Nature Research's author and referees' website at www.nature.com/authors for information about policies, services and author benefits**

Communications Psychology is committed to improving transparency in authorship. As part of our efforts in this direction, we are now requesting that all authors identified as 'corresponding author' create and link their Open Researcher and Contributor Identifier (ORCID) with their account on the Manuscript Tracking System prior to acceptance. ORCID helps the scientific community achieve unambiguous attribution of all scholarly contributions. You can create and link your ORCID from the home page of the Manuscript Tracking System by clicking on 'Modify my Springer Nature account' and following the instructions in the link below. Please also inform all co-authors that they can add their ORCIDs to their accounts and that they must do so prior to acceptance.
https://www.springernature.com/gp/researchers/orcid/orcid-for-nature-research

For more information please visit http://www.springernature.com/orcid

If you experience problems in linking your ORCID, please contact the Platform Support Helpdesk.

Version 1:

Decision Letter:

** Please ensure you delete the link to your author homepage in this e-mail if you wish to forward it to your coauthors **

Dear Ms Xu,

Your manuscript titled "Robust Evidence for Theta-Band Rhythmicity in Behavior across Two Dense-Sampling Datasets" has now been seen by our reviewers, whose comments appear below. In light of their advice I am delighted to say that we are happy, in principle, to publish a suitably revised version in Communications Psychology.

We therefore invite you to revise your paper one last time to address the remaining concerns of our reviewers and a list of editorial requests. At the same time we ask that you edit your manuscript to comply with our format requirements and to maximise the accessibility and therefore the impact of your work.

EDITORIAL REQUESTS:

Please review our specific editorial comments and requests regarding your manuscript in the attached "Editorial Requests Table". Please outline your response to each request in the right hand column. Please upload the completed table with your manuscript files as a Related Manuscript file.

If you have any questions or concerns about any of our requests, please do not hesitate to contact me.

SUBMISSION INFORMATION:

In order to accept your paper, we require the files listed here https://www.nature.com/documents/commsj-file-checklist.pdf .

OPEN ACCESS:

Communications Psychology is a fully open access journal. Articles are made freely accessible on publication. For further information about article processing charges, open access funding, and advice and support from Nature Research, please visit https://www.nature.com/commspsychol/open-access

At acceptance, you will be provided with instructions for completing the open access licence agreement on behalf of all authors. This grants us the necessary permissions to publish your paper. Additionally, you will be asked to declare that all required third party permissions have been obtained, and to provide billing information in order to pay the article-processing charge (APC).

* TRANSPARENT PEER REVIEW: Communications Psychology uses a transparent peer review system. On author request, confidential information and data can be removed from the published reviewer reports and rebuttal letters prior to publication. If you are concerned about the release of confidential data, please let us know specifically what information you would like to have removed. Please note that we cannot incorporate redactions for any other reasons.

* CODE AVAILABILITY: All Communications Psychology manuscripts must include a section titled "Code Availability" at the end of the methods section. We require that the custom analysis code supporting your conclusions is made available in a publicly accessible repository at this stage; please choose a repository that generates a digital object identifier (DOI) for the code; the link to the repository and the DOI must be included in the Code Availability statement. Publication as Supplementary Information will not suffice.

* DATA AVAILABILITY:
All Communications Psychology manuscripts must include a section titled "Data Availability" at the end of the Methods section. More information on this policy, is available in the Editorial Requests Table and at http://www.nature.com/authors/policies/data/data-availability-statements-data-citations.pdf.

Please use the following link to submit the above items:
Link Redacted
** This url links to your confidential home page and associated information about manuscripts you may have submitted or be reviewing for us. If you wish to forward this email to co-authors, please delete the link to your homepage first **

We hope to hear from you within two weeks; please let us know if you need more time.

Best regards,

Troby Lui

Troby Lui, PhD
Associate Editor
Communications Psychology

David Pascucci
Guest Editor
Communications Psychology

REVIEWERS' COMMENTS:

Reviewer #1 (Remarks to the Author):

The authors have addressed many of my concerns and the manuscript is much improved on several levels.
At this time - the only remaining point I noticed upon re-reading the revised manuscript is the following sentence in the abstract:

"However, identifying such a behavioral signature has remained controversial: evidence for behavioral oscillations is not yet firmly established, and aperiodic components can be misidentified as rhythmic"

I think that this paper makes an interesting a solid contribution to the literature - but it isn't the first to account for aperiodicity in behavioural oscillations. For example, if I am not mistaken, aperiodic components of the spectra were considered by, e.g., Re et al., 2023 J Con Neuro in their revised analysis of accuracy time courses. It ok to indicate there is controversy and room for a closer analytic consideration. But it probably is slightly overstated to say that evidence for behavioural oscillations is not yet firmly established -- and for this particular reason.

It is a small thing - and mostly relates to the culture of advancing knowledge rather than of true essence.

Reviewer #2 (Remarks to the Author):

I thank the authors for responding to my comments, the responses address all concerns.

Reviewer #3 (Remarks to the Author):

I thank the authors for their effort in addressing my and the other reviewers' comments, in particular for performing in-depth additional analyses. I am satisfied with the changes and have no further suggestions.

-Anthony Harris

** Visit Nature Research's author and referees' website at www.nature.com/authors for information about policies, services and author benefits**

Open Access This Peer Review File is licensed under a Creative Commons Attribution 4.0 International License, which permits use, sharing, adaptation, distribution and reproduction in any medium or format, as long as you give appropriate credit to the original author(s) and the source, provide a link to the Creative Commons license, and indicate if changes were made.
In cases where reviewers are anonymous, credit should be given to 'Anonymous Referee' and the source.
The images or other third party material in this Peer Review File are included in the article's Creative Commons license, unless indicated otherwise in a credit line to the material. If material is not included in the article's Creative Commons license and your intended use is not permitted by statutory regulation or exceeds the permitted use, you will need to obtain permission directly from the copyright holder.
To view a copy of this license, visit https://creativecommons.org/licenses/by/4.0/

Reviewer 1

General response

Thank you for all the insightful comments. We have made several revisions based on your suggestions, and we believe the revised manuscript is clearer and better motivated as a result. The main revisions are listed below:

(1) We revised the Introduction and Discussion to better motivate the study at the cognitive level, by connecting behavioral rhythmicity to theoretical accounts of dynamic prioritization in perception and attention.

(2) We revised the title to more accurately reflect the scope of the study. The new title is: " Robust Evidence for Theta-Band Rhythmicity in Behavior across Two Dense-Sampling Datasets."

(3) We added a schematic diagram of the participant-level AR-surrogate analysis pipeline to make the method easier to follow.

(4) We added an example single-participant figure showing accuracy and RT time courses across ISIs, providing a more intuitive view of the behavioral data entering the spectral analysis.

(5) We added the eye-tracking-related trial exclusion statistics for Experiment 2 and included a supplementary figure showing participant-level exclusion rates.

(6) We corrected the tilt-report diagram so that clockwise and counter-clockwise tilts relative to the vertical axis are labeled correctly.

(7) We revised the relevant figures to show statistical outcomes more directly, including significance markers for the peak-frequency and behavioral-performance panels.

Comment 1

Although the paper in essence is a reanalysis and attempted replication – I think that the authors can do a bit better in fully motivating the study. Generally, the introduction and the discussion prioritize linking the work to neuronal oscillations touching on a broad gamut of whether those are or aren't relevant. I wonder whether the literature on cognitive models of sensory processing and response selection might make for a more natural background for the work. Of course considering the behavioral rhythms, those could link to models on how and why such cognitive functions might engage in fluctuations (Fiebelkorn et al., 2019 as well as Re et al., 2025 provide nice theoretical frameworks for those).

Response

We thank the reviewer for this helpful suggestion. In the revised Introduction, we have broadened the opening theoretical framework by linking the oscillatory-segregation account to cognitive-level accounts of temporally structured perception and attention (Page 2; Lines 29-33): "A related challenge is how competing sensory and action-relevant information is prioritized over time. Recent theoretical accounts suggest that perception and attention may operate through dynamic fluctuations rather than continuous processing, allowing task-relevant information to be periodically prioritized while the system remains sensitive to new input[5,6]."

In addition, in the revised Discussion, we further connect this cognitive framing to the present task (Page 13; Lines 512-518): "Functionally, this temporal organization may help coordinate sensory selection and response preparation[5,6], allowing selected sensory input to be linked to the appropriate response while competing mappings are kept apart. In our paradigm, participants had to select one of two sensory inputs and map it onto a left- or right-hand response. Because all information will be partially activated across trials, temporal binding of the currently-relevant task processes (e.g., for cue LR, left stimulus and right hand) via rhythmic alternation can help reduce representational interference and help to address the binding problem[41,42]."

Comment 2

The title of the paper seems to promise a bit more than what thie report actually is. Rhythmicitiy isn't quite revisited here. The paper provides a validation of previous findings rather than challenge. Re-evaluation if anything in the most literal sense rather than a revisitation that sound more provocative than what this actually delivers.

Response

We agree that the original title sounded somewhat more provocative than intended. We have therefore revised the title to better reflect the scope of the study. The revised title is: "Robust Evidence for Theta-Band Rhythmicity in Behavior across Two Dense-Sampling Datasets."

Comment 3

Including a diagram of the analysis pipeline would be lovely. Indeed, the paper referenced has such a diagram, however, including a similar diagram here would make a good educational point – which would add value to this publication.

Response

We agree that a schematic overview would improve clarity. We have therefore added a diagram of the AR-surrogate analysis pipeline (Fig. 2), illustrating the steps from participant-level behavioral time courses to deviation spectra and group-level statistics.

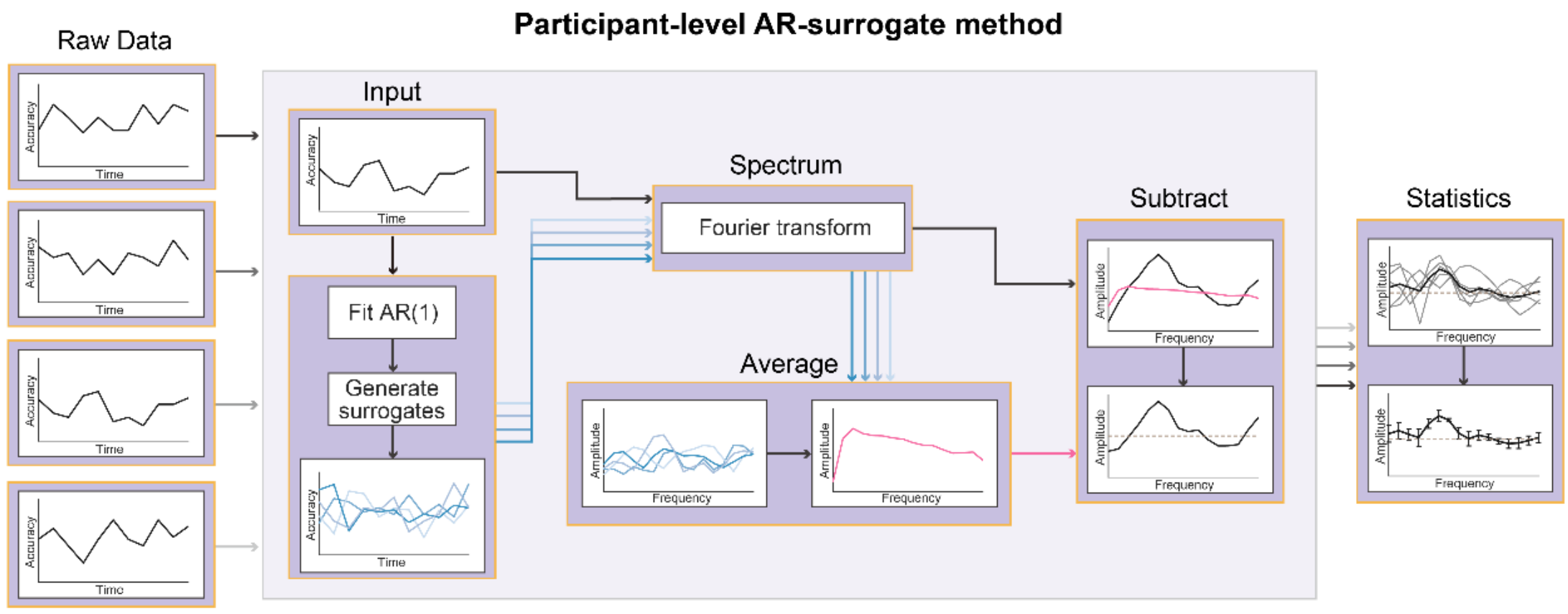

Figure 2. Analysis pipeline for participant-level AR-surrogate analyses. Participant-level behavioral time courses were constructed from accuracy or response time across cue–target intervals. An AR(1) model was fitted to each empirical time course to generate surrogate series capturing aperiodic temporal structure. Empirical and surrogate series were then submitted to Fourier analysis, and the average surrogate spectrum was subtracted from the empirical spectrum to obtain a deviation spectrum. Group-level statistics were applied to the deviation spectra to identify reliable rhythmic components. The figure is adapted from Harris and Beale (2025).

Comment 4

inclusion of time series data. If possible it would be really nice to see examples of accuracy and RT time courses. I realize that averaging those for the group might be tricky considering the approach (and perhaps the nature of the data). But having an example subject would be a nice addition for the readers to gain intuitive sense of what the spectra consist of.

Response

We have added a single-participant figure showing accuracy and RT time courses across ISIs for both experiments, using Subject 1 as the example (Fig. S1).

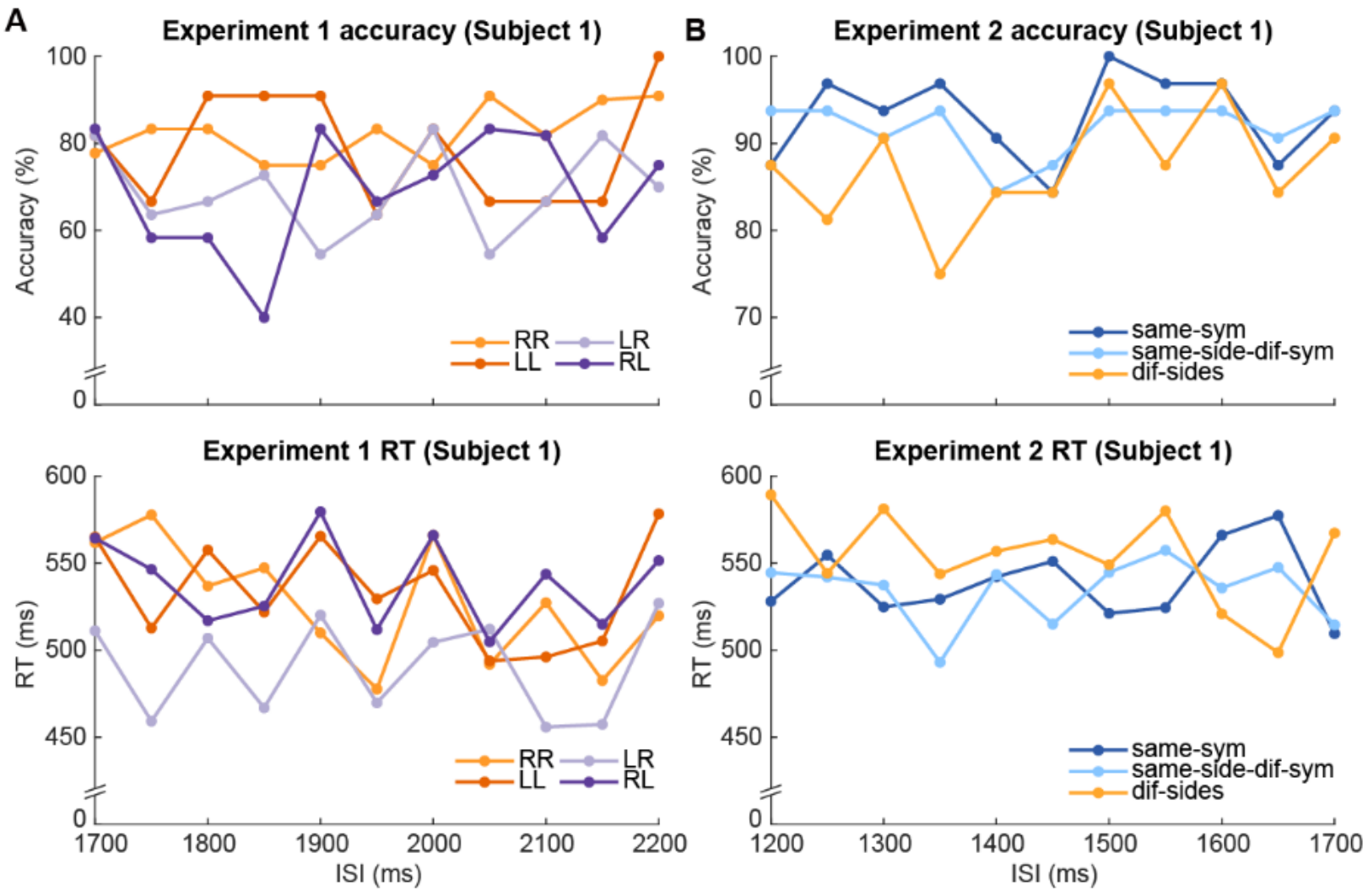

Figure S1. Example single-participant behavioral time courses. (A) Accuracy and RT time courses across ISIs for Subject 1 from Experiment 1. (B) Accuracy and RT time courses across ISIs for Subject 1 from Experiment 2. These time courses illustrate the behavioral data entered into the AR-surrogate spectral analysis.

Comment 5

Fourteen participants were removed due to more than 50% trial exclusion in experiment 2 (due to eye movements). What are the stats of eye movements in the included participants?

Response

We have now added the eye-tracking-related trial exclusion statistics for Experiment 2 (Page 10; Lines 373–377) and included a supplementary figure showing the exclusion rate for each screened participant (Fig. S2): "As part of this screening, participants were excluded when more than 50% of their trials were rejected based on the eye-tracking criteria. Across all screened participants, the eye-tracking-related trial exclusion rate was 39.30% ± 29.60% (mean ± SD; range = 2.42%–97.94%). Among the included participants, this rate was 21.47% ± 16.42% (range = 2.42%–49.91%; Fig. S2)."

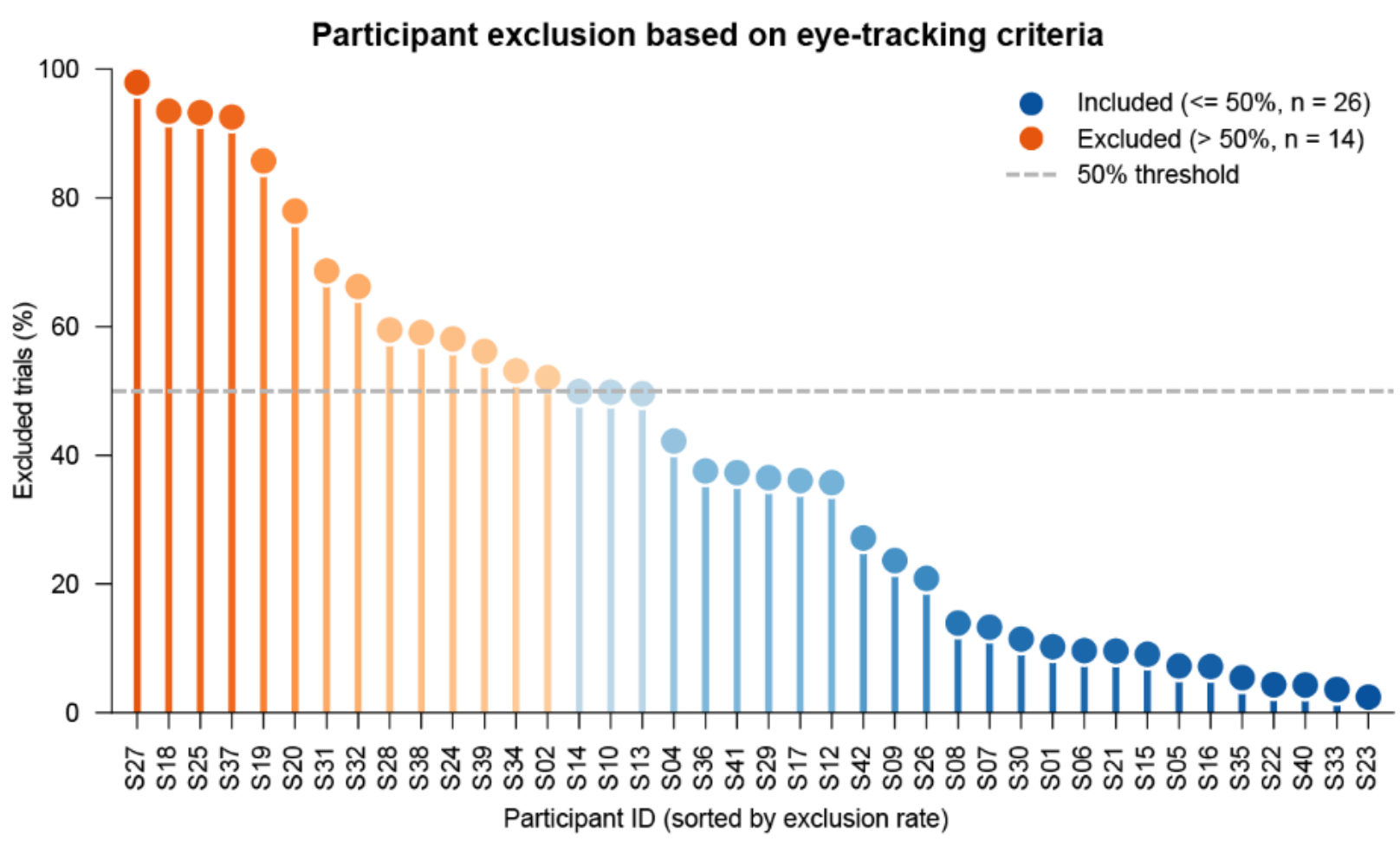

Figure S2. Eye-tracking-related trial exclusion rate in Experiment 2. Each point shows the subject-level mean percentage of trials excluded by the eye-tracking criteria across sessions. Participants are sorted from highest to lowest exclusion rate. Orange points indicate participants excluded by the predefined criterion (>50% trials rejected), and blue points indicate participants retained for analysis (≤50% trials rejected). The dashed line marks the 50% exclusion threshold.

Comment 6

The diagram for tilt report is either confusing or erroneous. It looks like its labeled opposite than what I would deem CW or CCW tile off vertical

Response

We thank the reviewer for pointing this out. We have corrected the tilt-report diagram to ensure that clockwise and counter-clockwise tilts relative to the vertical axis are labeled correctly.

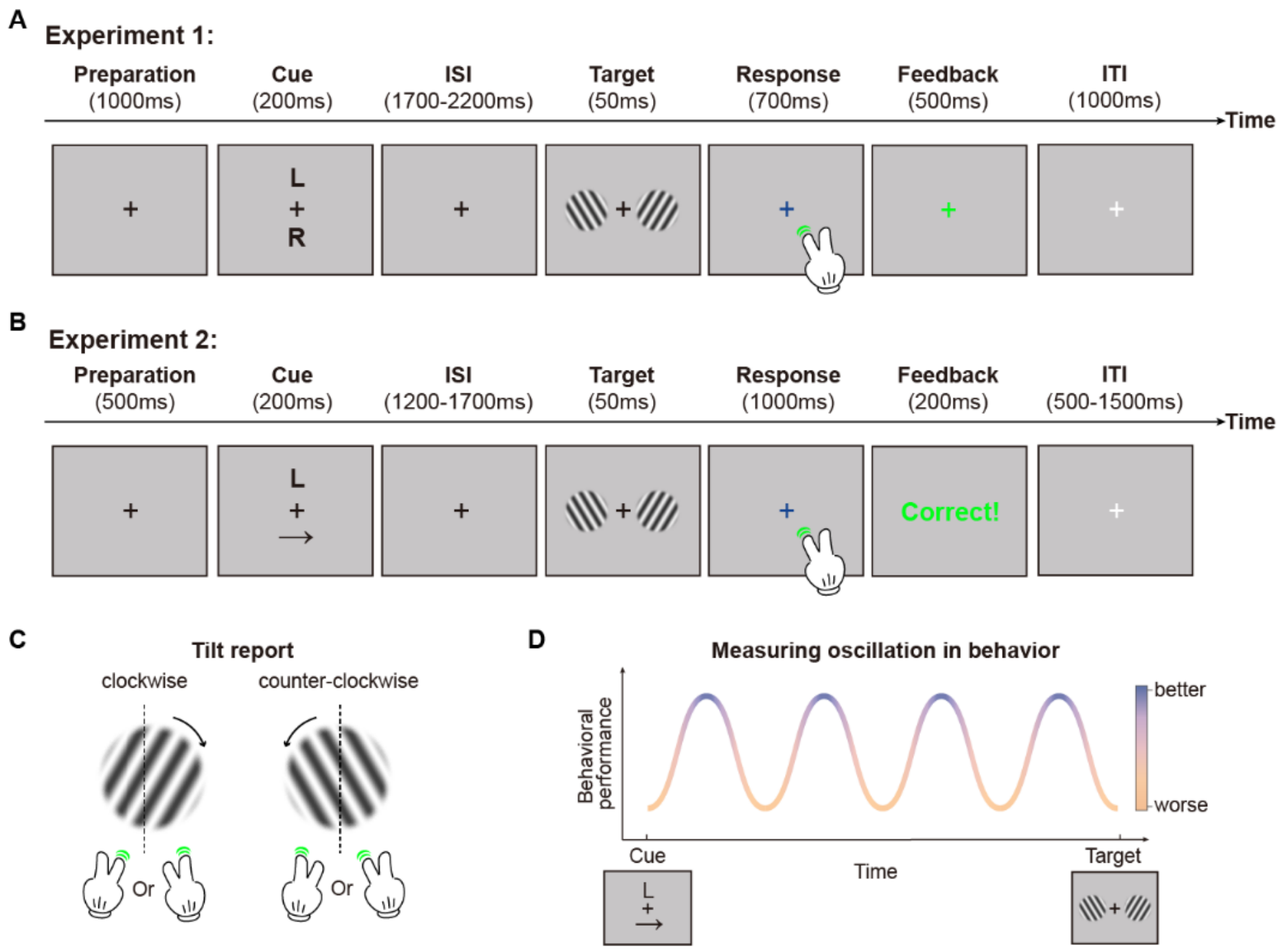

Figure 1. Paradigm. (A) Illustration of a single trial in Experiment 1. (B) Illustration of a single trial in Experiment 2. (C) Task display. Participants reported whether the target grating was tilted clockwise or counter-clockwise relative to vertical, using the index or middle finger of the cued hand. (D) Behavioral performance (accuracy/reaction time) was sampled across cue–target intervals, yielding a time course on which rhythmic fluctuations can be assessed.

Comment 7

Might the authors consider indicating significance to the peak frequency barplot as well?

Response

We have revised the relevant figures, including Figs. 3, 4, and 6, by adding significance markers where condition comparisons were shown.

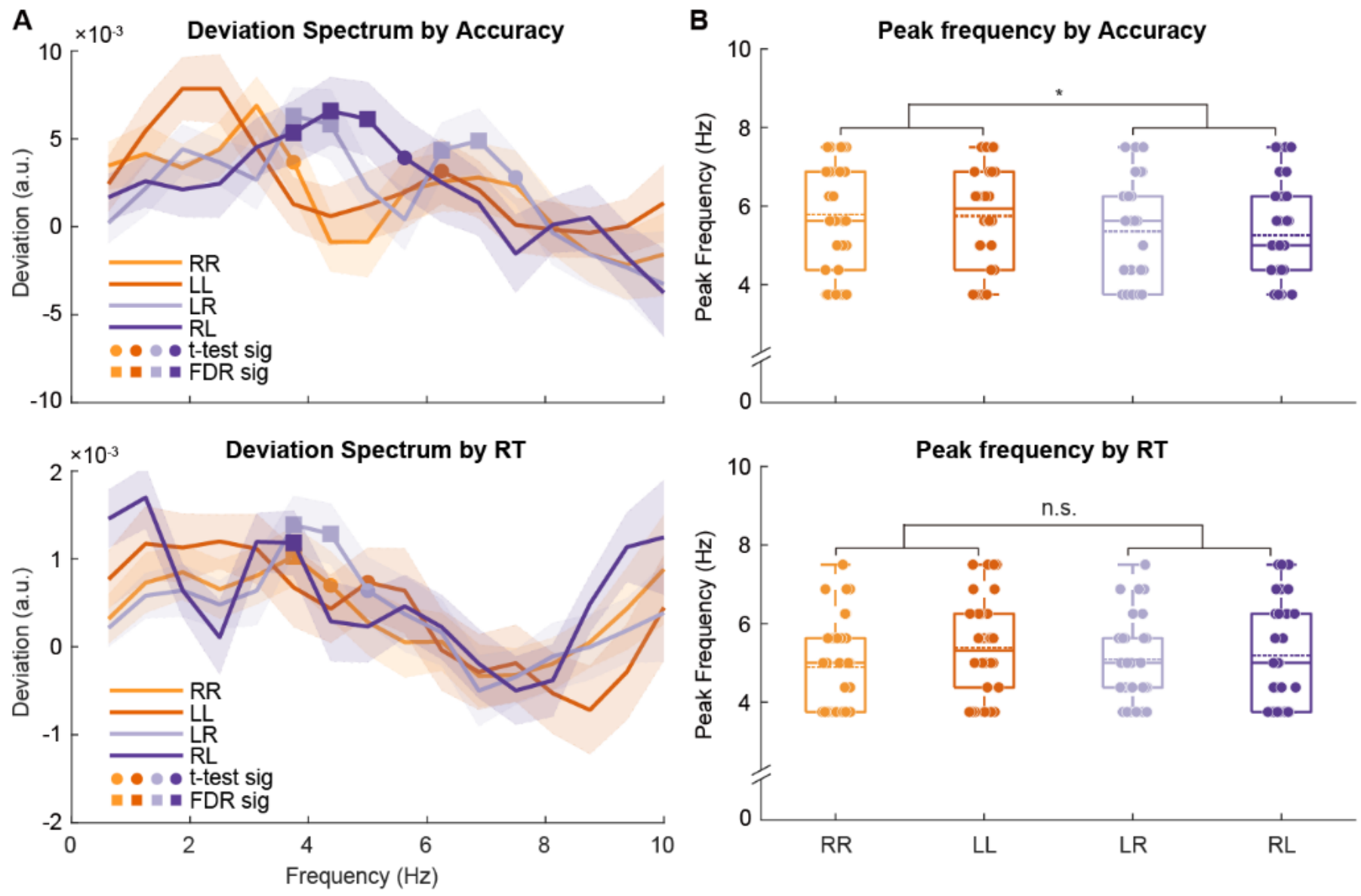

Figure 3. Results of Experiment 1 (n = 34 participants). (A) Group-mean deviation spectra for accuracy (top) and reaction time (bottom). Curves correspond to the four conditions (RR, LL, LR, RL); shaded areas indicate ± s.e.m. Filled circles mark frequencies significant by one-tailed one-sample t-tests across participants ($p < 0.05$); filled squares mark frequencies surviving Benjamini–Hochberg FDR correction across frequencies ($q = 0.05$). Statistical analysis was restricted to the theta-frequency range (3.75 - 7.50 Hz). (B) Distributions of individual theta peak frequencies for accuracy (top) and reaction time (bottom). Box plots show the median and interquartile range (whiskers, 1.5 × IQR); points indicate individual participants; solid horizontal lines indicate the mean peak frequency. * indicates $p < .05$; n.s. indicates not significant.

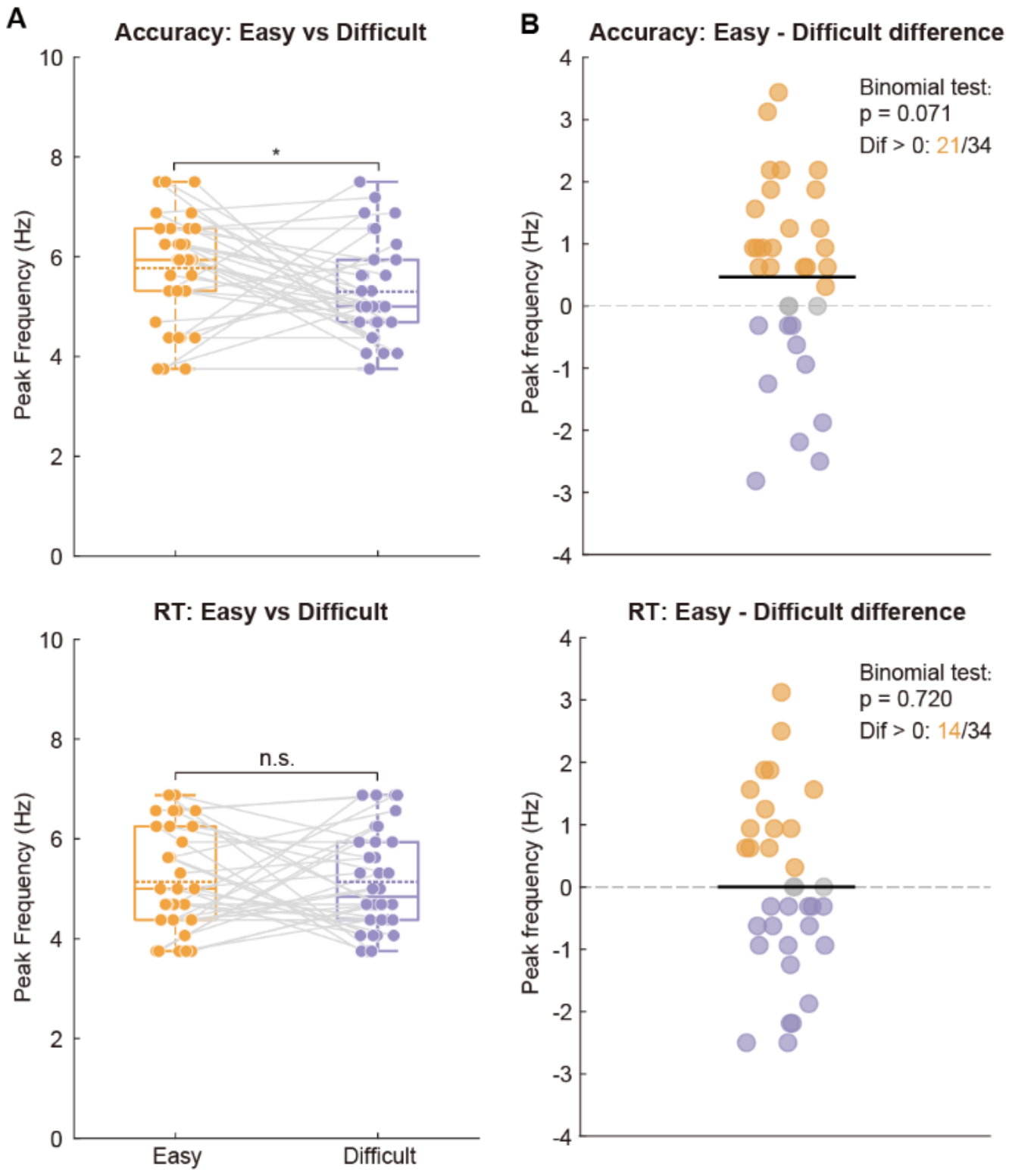

Figure 4. Peak frequency differences between easy and difficult rules (n = 34 participants). (A) Peak frequency for easy rules (RR and LL) and difficult rules (LR and RL), shown separately for accuracy-based estimates (top) and RT-based estimates (bottom). Dots represent individual participants, grey lines connect paired observations from the same participant, and boxplots show the group distributions. The dashed horizontal lines within each box indicate the condition means. (B) Participant-level easy-minus-difficult differences in peak frequency, shown separately for accuracy-based estimates (top) and RT-based estimates (bottom). Positive values indicate higher peak frequency for easy than difficult rules. The black horizontal line indicates the group mean difference, and the grey dashed line marks zero. Orange dots indicate participants with positive differences, purple dots indicate negative differences, and grey dots indicate zero differences. The inset reports the binomial test for the number of participants showing a positive difference. * indicates $p < .05$; n.s. indicates not significant.

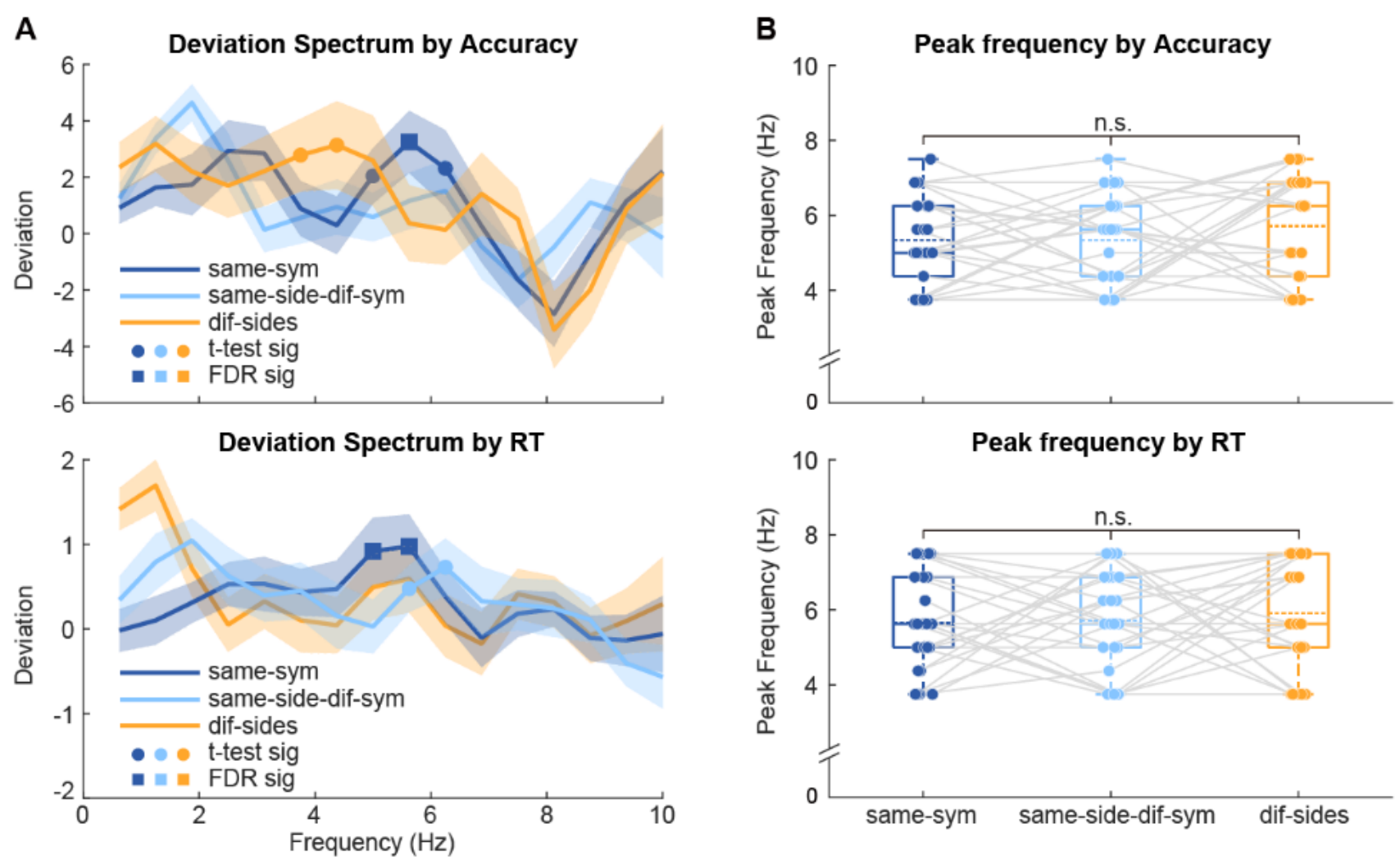

Figure 6. Results of Experiment 2 (n = 26 participants). (A) Group-mean deviation spectra for accuracy (top) and reaction time (bottom). Curves correspond to the three conditions (same-sym, same-side-dif-sym, and dif-sides); shaded areas indicate ± s.e.m. Filled circles mark frequencies significant by one-tailed one-sample t-tests across participants ($p < 0.05$); filled squares mark frequencies surviving Benjamini–Hochberg FDR correction across frequencies ($q = 0.05$). Statistical analysis was restricted to the theta-frequency range (3.75 - 7.50 Hz). (B) Distributions of individual theta peak frequencies for accuracy (top) and reaction time (bottom). Box plots show the median and interquartile range (whiskers, 1.5 × IQR); points indicate individual participants; solid horizontal lines indicate the mean peak frequency. n.s. indicates not significant.

Reviewer 2

General response

Thank you for all the insightful comments. We have revised the manuscript to clarify the role of the Experiment 1 reanalysis, add participant-level information, and improve the statistical and visual presentation. The main revisions are listed below:

(1) We clarified that the Experiment 1 reanalysis serves as a robustness check of the previously reported findings and helps interpret Experiment 2

(2) We added participant-level summaries of behavioral rhythmicity across experiments, conditions, and measures, now reported in Table S1.

(3) We expanded the participant-level peak-frequency analysis by adding easy–difficult difference plots and reporting the number of participants showing the predicted direction.

(4) We revised the peak-frequency figures to show raw frequencies in Hz instead of z-scored values, and added paired lines to make within-participant changes across conditions easier to see.

(5) We revised the Discussion to more carefully interpret the weaker and less consistent rhythmicity pattern in Experiment 2, including the possibility that the revised cue design and limited observations per condition increased variability in condition-level time courses.

(6) We clarified why the statistical analyses focused on the theta-frequency range and explained that the exact range reflected the discrete frequency bins produced by the FFT procedure.

(7) We improved the readability of figures by replacing abbreviated condition labels with more explicit labels and by writing out tilt-response labels where appropriate.

(8) We clarified the structure of the Experiment 1 dataset, including the approximate number of trials and the fact that the original dataset included behavioral, eye-tracking, and EEG recordings, although the present reanalysis focused on behavioral data.

Comment 1

The reanalysis of the dataset #1 confirms the previous results. But do we learn more from the improved analysis? Does it have any added value?

Response

We thank the reviewer for raising this important point. We agree that the reanalysis of dataset 1 mainly confirms the previously reported pattern. However, we believe that this confirmation is still informative, especially given the current debate about behavioral oscillations.

First, our analysis establishes the existence of behavioral oscillations in this earlier data set; the earlier report assumed such behavioral oscillations, and then compared them across conditions. Given the debate about the existence of behavioral oscillations, this is already a useful contribution. Second, a major concern in this field is that apparent behavioral rhythms may sometimes reflect aperiodic temporal structure or other non-rhythmic dependencies rather than genuine oscillatory activity. By applying a participant-level AR-surrogate approach, the present reanalysis shows that the previously reported theta-band rhythmicity and difficulty-related frequency shift remain detectable after controlling for aperiodic temporal structure. This strengthens the interpretation that the original finding is unlikely to be explained simply by the original spectral method or by aperiodic dynamics.

The reanalysis also helps interpret dataset 2. Because the improved AR-based pipeline recovered the previously reported frequency shift in dataset 1, the absence of a reliable shift in dataset 2 is less likely to reflect a limitation of the analysis approach itself. Instead, it suggests that frequency modulation by task difficulty requires further support, whereas theta-band rhythmicity in behavior appears more robust.

We have revised the Discussion to clarify this point (Page 13; Lines 525 -527): "The successful recovery of the previously reported frequency shift in Experiment 1 helps frame the null

frequency-shift result in Experiment 2. It suggests that the absence of a reliable shift in Experiment 2 should not be attributed to the analysis approach itself."

Comment 2

At the moment, the authors present group-level results, but as I understand the method, also individual level statements can be made, e.g. how many participants show significant behavioral rhythmicity, are there shifts on the individual level, etc. Given the usual interindividual variability in such data, I think it would be great to include also more analysis on the individual level.

Response

In the revised manuscript, we now provide a clearer participant-level description of the results, complementing the group-level analyses. Specifically, we made three changes.

First, to describe how many participants showed behavioral rhythmicity, we added a descriptive participant-level summary. For each participant, condition, and measure, behavioral rhythmicity was defined as the presence of at least one positive deviation value within the theta-frequency range. Using this criterion, rhythmicity was observed in most participants across conditions and measures. In Experiment 1, 33–34 of 34 participants showed rhythmicity across the four conditions for both accuracy and RT. In Experiment 2, rhythmicity was observed in 24–26 of 26 participants across the three conditions for accuracy, and in 25–26 of 26 participants for RT. The full condition-wise counts are now reported in Table S1.

Table S1. *Number of participants showing at least one positive theta-band deviation.*

Experiment	Measure	Condition	Rhythmic Count
Experiment 1	Accuracy	RR	33/34
		LL	34/34
		LR	34/34
		RL	34/34
	RT	RR	33/34
		LL	33/34
		LR	34/34
		RL	34/34
Experiment 2	Accuracy	same-sym	26/26
		same-side-dif-sym	25/26
		dif-sides	24/26
	RT	same-sym	25/26
		same-side-dif-sym	26/26
		dif-sides	25/26

Note. Participants were counted as showing behavioral rhythmicity if their deviation spectrum showed at least one positive value in the theta range (3.75–7.50 Hz). Counts indicate the number of participants meeting this criterion out of the total number of participants.

Second, we expanded the frequency-shift analysis to include the participant-level direction of the easy–difficult difference. To make this clearer, we added Fig. 4, which presents the easy–difficult peak-frequency comparison in Experiment 1 separately for accuracy-based and RT-based estimates. Fig. 4A shows the paired easy and difficult peak-frequency estimates for each participant, with grey lines connecting observations from the same participant. Fig. 4B shows the corresponding easy-minus-difficult difference scores, making it easier to see how many participants showed the predicted direction and how large these individual differences were. We also revised the Results section to report these participant-level directional patterns alongside the group-level LMM results (Page 9; Lines 345-364):"Consistent with the original report, in accuracy, the 2 × 2 linear mixed-effects model showed a significant hand × target-location interaction (Type-III ANOVA with Satterthwaite df: $F_{(1, 99)}$ = 4.25, p = .042, η_p^2 = .04, 95% CI [.00, .14]; Fig. 3B, top). To directly test the effect of rule difficulty, we additionally compared easy rules (RR and LL) with difficult rules (LR and RL). This follow-up linear mixed-effects model confirmed that peak theta frequency was lower for difficult rules than for easy rules ($F_{(1, 101)}$ = 4.33, p = .040, η_p^2 = .04, 95% CI [.00, .14]; Fig. 4A, top). At the participant level, 21 of 34 participants showed a higher accuracy-based peak frequency for easy than difficult rules, indicating a marginal trend consistent with the group-level effect (binomial test, p = .071; Fig. 4B, top).

Parallel analyses using RT-based peak estimates did not show the same pattern. The 2 × 2 linear mixed-effects model did not provide significant evidence for a hand × target-location interaction ($F_{(1, 99)}$ < 0.01, p = 1.000, η_p^2 = .00, 95% CI [.00, .00]; Fig. 3B, bottom). A Bayesian model comparison further provided moderate evidence against including this interaction (BF_{01} = 3.98). The corresponding easy-difficult comparison was also not significant (Fig. 4A, bottom), with only 14 of 34 participants showing the easy > difficult direction for RT-based peaks (binomial test, p = .720; Bayesian paired t-test: BF_{01} = 5.44; Fig. 4B, bottom)."

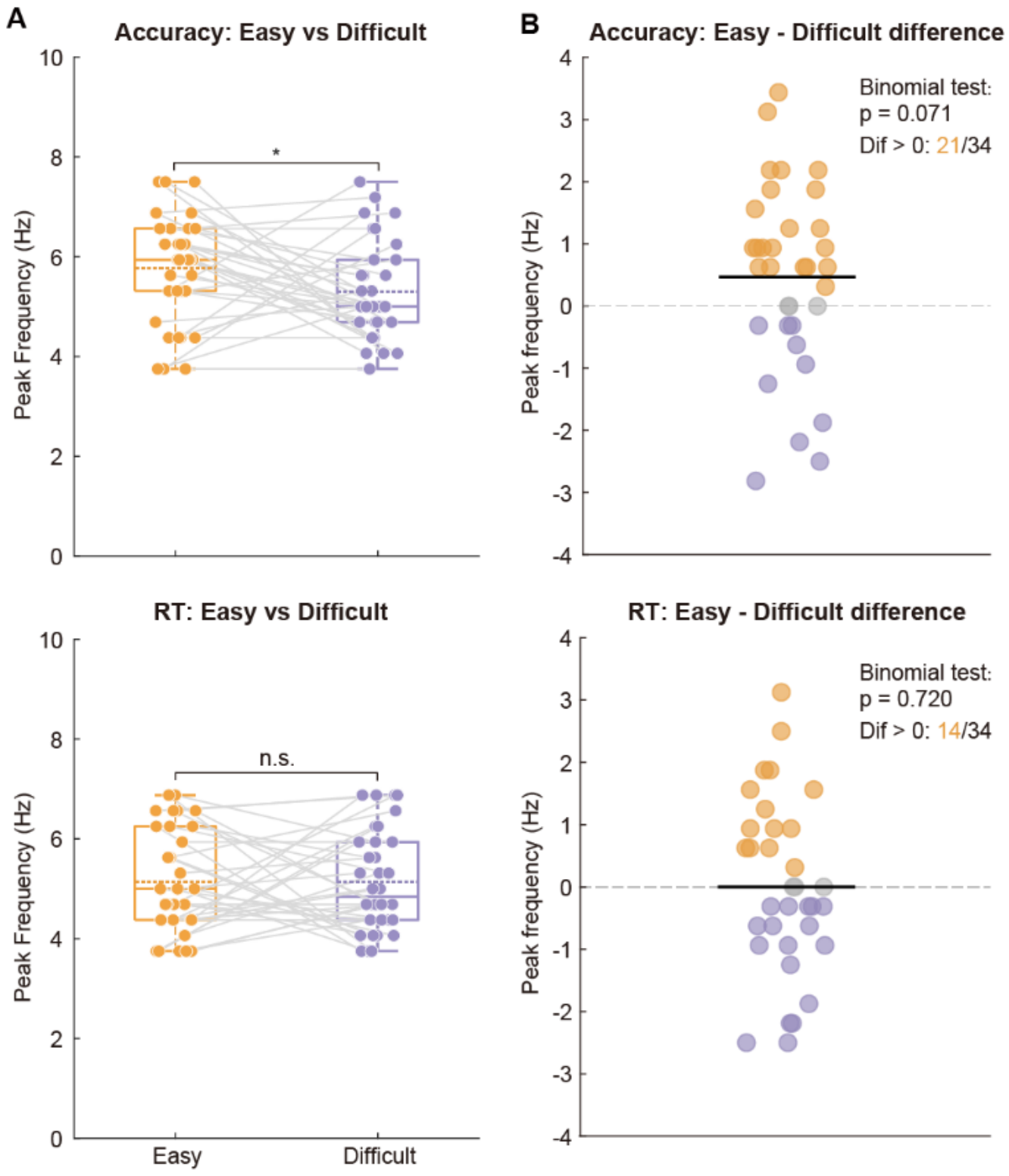

Figure 4. Peak frequency differences between easy and difficult rules (n = 34 participants). (A) Peak frequency for easy rules (RR and LL) and difficult rules (LR and RL), shown separately for accuracy-based estimates (top) and RT-based estimates (bottom). Dots represent individual participants, grey lines connect paired observations from the same participant, and boxplots show the group distributions. The dashed horizontal lines within each box indicate the condition means. (B) Participant-level easy-minus-difficult differences in peak frequency, shown separately for accuracy-based estimates (top) and RT-based estimates (bottom). Positive values indicate higher peak frequency for easy than difficult rules. The black horizontal line indicates the group mean difference, and the grey dashed line marks zero. Orange dots indicate participants with positive differences, purple dots indicate negative differences, and grey dots indicate zero differences. The inset reports the binomial test for the number of participants showing a positive difference. * indicates $p < .05$; n.s. indicates not significant.

Third, we revised the visual presentation of the peak-frequency results to make within-participant patterns easier to follow. In Fig. 4, the grey lines connect each participant's easy and difficult estimates, so the direction and size of the easy–difficult difference can be seen directly. We used the same logic in Fig. 6 by adding connecting lines across the three conditions in Experiment 2, allowing readers to see how each participant's peak-frequency estimates change across conditions rather than only comparing the group distributions.

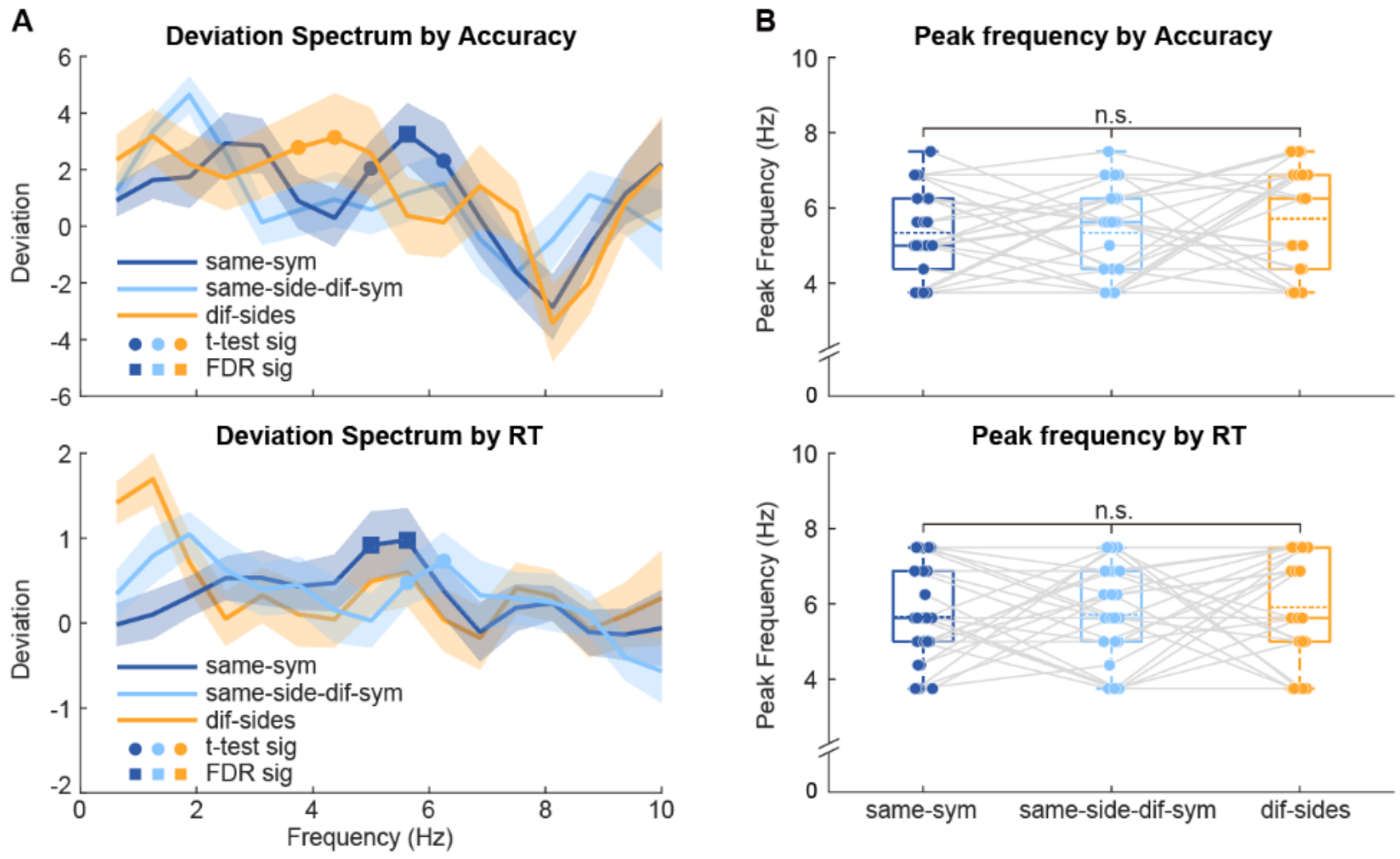

Figure 6. Results of Experiment 2 (n = 26 participants). (A) Group-mean deviation spectra for accuracy (top) and reaction time (bottom). Curves correspond to the three conditions (same-sym, same-side-dif-sym, and dif-sides); shaded areas indicate ± s.e.m. Filled circles mark frequencies significant by one-tailed one-sample t-tests across participants ($p < 0.05$); filled squares mark frequencies surviving Benjamini–Hochberg FDR correction across frequencies ($q = 0.05$). Statistical analysis was restricted to the theta-frequency range (3.75 - 7.50 Hz). (B) Distributions of individual theta peak frequencies for accuracy (top) and reaction time (bottom). Box plots show the median and interquartile range (whiskers, 1.5 × IQR); points indicate individual participants; solid horizontal lines indicate the mean peak frequency. n.s. indicates not significant.

Comment 3

The behavioral rhythms do not seem to be very consistent, as dataset #2 seems to indicate that behavioral rhythms cannot be detected reliably across all conditions. It's also tricky to say because the conditions are not compared directly. But the reader is left wondering if and why this is the case, as the discussion simply states that the authors "again observed theta-band rhythmicity in behavioral performance" for experiment #2, without further discussion of the potential differences between the two datasets. This does not seem like a sufficient analysis and discussion of the results.

Response

The reviewer is right that the rhythmicity pattern in Experiment 2 was not as uniform as in Experiment 1, and our previous Discussion did not make this contrast sufficiently clear. We have therefore revised the Discussion to describe the Experiment 2 results more cautiously.

In the revised manuscript, we now state more explicitly that Experiment 2 provided evidence for theta-band rhythmicity, but that this evidence was less consistent across conditions and measures than in Experiment 1. Specifically, we revised the first paragraph of the Discussion as follows (Page 12; Lines 497-501): "In Experiment 2, we used a modified cue design that separated cue ambiguity from task difficulty (Fig. 1B). This dataset[32] also showed evidence for theta-band rhythmicity in behavioral performance, although the pattern was weaker than in Experiment 1 (Fig. 6A). Moreover, unlike Experiment 1, Experiment 2 showed no reliable

frequency shifts as task demands changed (Fig. 6B)."

We also expanded the Discussion to provide a more careful interpretation of the weaker condition-wise pattern observed in Experiment 2. Specifically, we now discuss that the revised cue design and the limited number of observations per condition may have increased variability in condition-level time courses, which in turn could affect the precision of peak-frequency estimates (Page 13-14; Lines 525-549): "The successful recovery of the previously reported frequency shift in Experiment 1 helps frame the null frequency-shift result in Experiment 2. It suggests that the absence of a reliable shift in Experiment 2 should not be attributed to the analysis approach itself. Instead, the discrepancy between the two experiments points to possible limits in estimating behavioral frequency modulation. Indeed, whereas neural recordings provide substantial evidence for rhythmicity and frequency modulation[17,18,43–47], their behavioral signatures are harder to estimate reliably. In Experiment 2, theta-band rhythmicity was present, but it was less consistently expressed across conditions and measures than in Experiment 1. One reason may be that the revised cue design, while useful for separating cue ambiguity from task difficulty, also required each condition type to be pooled across several cue combinations. This may have made the behavioral time courses more heterogeneous than in the original paradigm. Moreover, although dense-sampling designs are powerful for detecting rhythmicity[26,48,49], the number of observations per participant remains limited once trials are divided by condition and interval. Thus, the weaker rhythmicity in some Experiment 2 conditions should not be interpreted as evidence that behavioral theta rhythms were absent in those conditions. Rather, it may reflect greater variability in condition-level time courses arising from both the pooling across cue combinations and the limited number of observations per interval.

This issue also carries over to the frequency-shift analysis. Detecting a shift in peak frequency requires stable rhythmic estimates within each condition before those estimates can be compared across task demands. If rhythmicity is weaker or more variable in some conditions, the extracted peak frequencies will also be less precise. Therefore, the null result of the frequency-shift analysis in Experiment 2 does not rule out frequency modulation; it may instead reflect insufficient within-participant data or greater within-condition variability, making it difficult to estimate condition-specific peaks with confidence."

Comment 4

Fig 2: It would be great to illustrate the procedure of the method in more detail. e.g. showing the process + individual level data in Fig. 2. This could make the method more accessible to readers who are not familiar with it, without having to consult Harris & Beale (2025) for details, and could also illustrate data quality.

Response

This point is closely related to a comment raised by reviewer 1, and we addressed both through the same revisions. We expanded the visual presentation of the AR-surrogate procedure to make the analysis pipeline easier to follow. Figure 2 now shows the main steps of the participant-level analysis, from behavioral time courses to AR(1) model fitting and surrogate

generation, Fourier analysis, computation of the deviation spectrum, and group-level statistics.

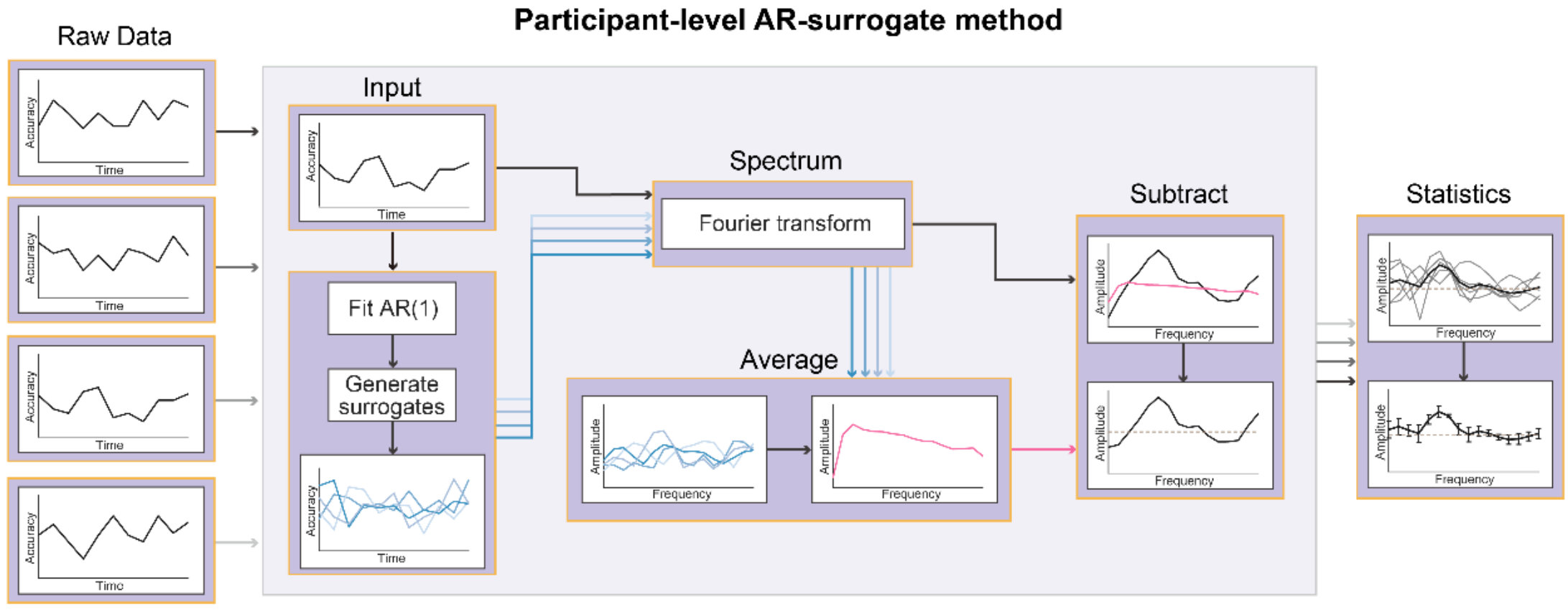

Figure 2. Analysis pipeline for participant-level AR-surrogate analyses. Participant-level behavioral time courses were constructed from accuracy or response time across cue–target intervals. An AR(1) model was fitted to each empirical time course to generate surrogate series capturing aperiodic temporal structure. Empirical and surrogate series were then submitted to Fourier analysis, and the average surrogate spectrum was subtracted from the empirical spectrum to obtain a deviation spectrum. Group-level statistics were applied to the deviation spectra to identify reliable rhythmic components. The figure is adapted from Harris and Beale (2025).

We also added Fig. S1, which shows example single-participant accuracy and RT time courses across ISIs for both experiments, using Subject 1 as the example. This figure illustrates the individual-level behavioral data entered into the AR-surrogate analysis and gives readers a clearer sense of the structure of the underlying time courses.

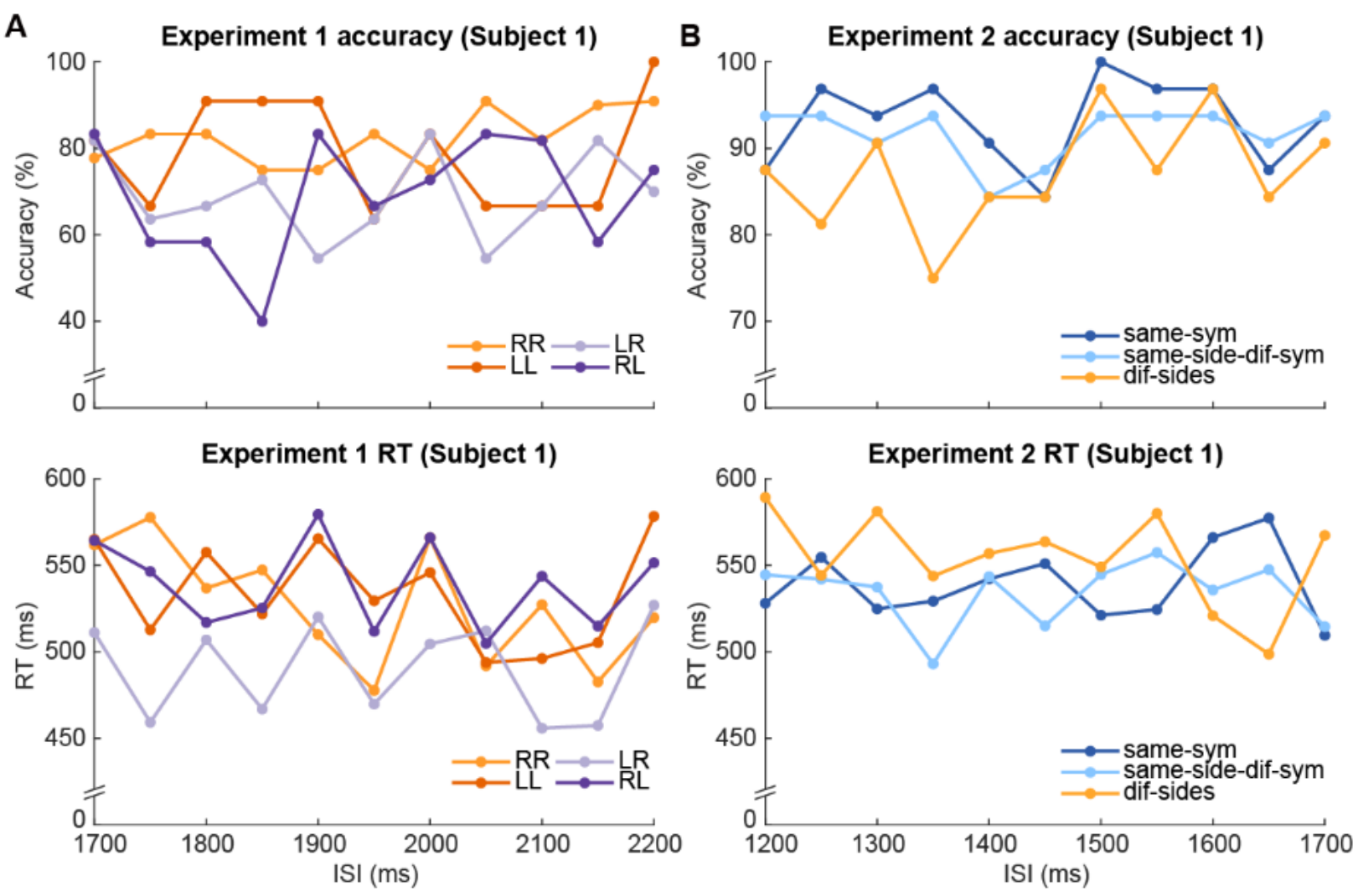

Figure S1. Example single-participant behavioral time courses. (A) Accuracy and RT time courses across ISIs for Subject 1 from Experiment 1. (B) Accuracy and RT time courses across ISIs for Subject

1 from Experiment 2. These time courses illustrate the behavioral data entered into the AR-surrogate spectral analysis.

Comment 5

The analysis was done for RT & accuracy in conjunction. Are there different expectations in terms of rhythmicity for these two measures? E.g. Fig. 2A shows quite an increase at about 10 Hz for the deviation spectrum for RT, that is cropped out in the analysis, as the "statistical analysis was restricted to theta-frequency range (3.75 - 7.50 Hz)" (also this seems to be a deviation from the theta-band range stated in the introduction; why?).

Response

We analyzed both accuracy and RT because they are the two main behavioral measures in this task, but we did not assume that they should show identical rhythmic profiles. Our main expectation concerned accuracy, because the original study reported the difficulty-related theta-frequency shift for accuracy-based peak estimates. RT was included as a complementary measure to provide a more complete assessment of behavioral rhythmicity, although the original study did not report a corresponding frequency-shift effect for RT. Indeed, it is well known that due to the speed-accuracy tradeoff, an effect may shift from expression in one dependent variable to the other, depending on a subject's position on this continuum. It is therefore worthwhile to consider the complementary variable RT.

The focus on theta was theory-driven. The present study was designed to test theta-band behavioral rhythmicity and to re-evaluate the previously reported theta-frequency shift. Accordingly, all statistical tests and multiple-comparison corrections were restricted to the theta-frequency range. The exact range used for statistical testing, 3.75–7.50 Hz, reflects the discrete frequency bins produced by the FFT procedure. Because these bins did not align exactly with the conventional 4–8 Hz theta range, we selected the available bins closest to that range.

The apparent increase around 10 Hz in the RT deviation spectrum falls outside the predefined theta range and was therefore not included in the statistical tests or multiple-comparison correction. Although this pattern may be worth exploring in future work, it appeared near the edge of the plotted frequency range, where estimates are less stable and harder to interpret. We therefore do not interpret it here to avoid overestimating its importance.

Comment 6

Fig 2B/4B: why is the frequency z-scored? Maybe it would be more informative to show the actual frequencies. Also possibly show the paired relationship between the conditions.

Response

In the previous version, peak frequencies were z-scored within each participant to emphasize relative differences across conditions. However, we agree that raw frequencies in Hz are more directly interpretable for evaluating peak-frequency shifts.

We therefore revised all relevant peak-frequency figures (Figs. 3, 4, and 6) to show raw peak frequencies in Hz rather than z-scored values, and updated the corresponding statistical analyses to use raw peak-frequency estimates. The results remained similar before versus after z-scoring. We also added paired lines connecting observations from the same participant across conditions, so that the within-participant direction and magnitude of the frequency differences can be seen more directly.

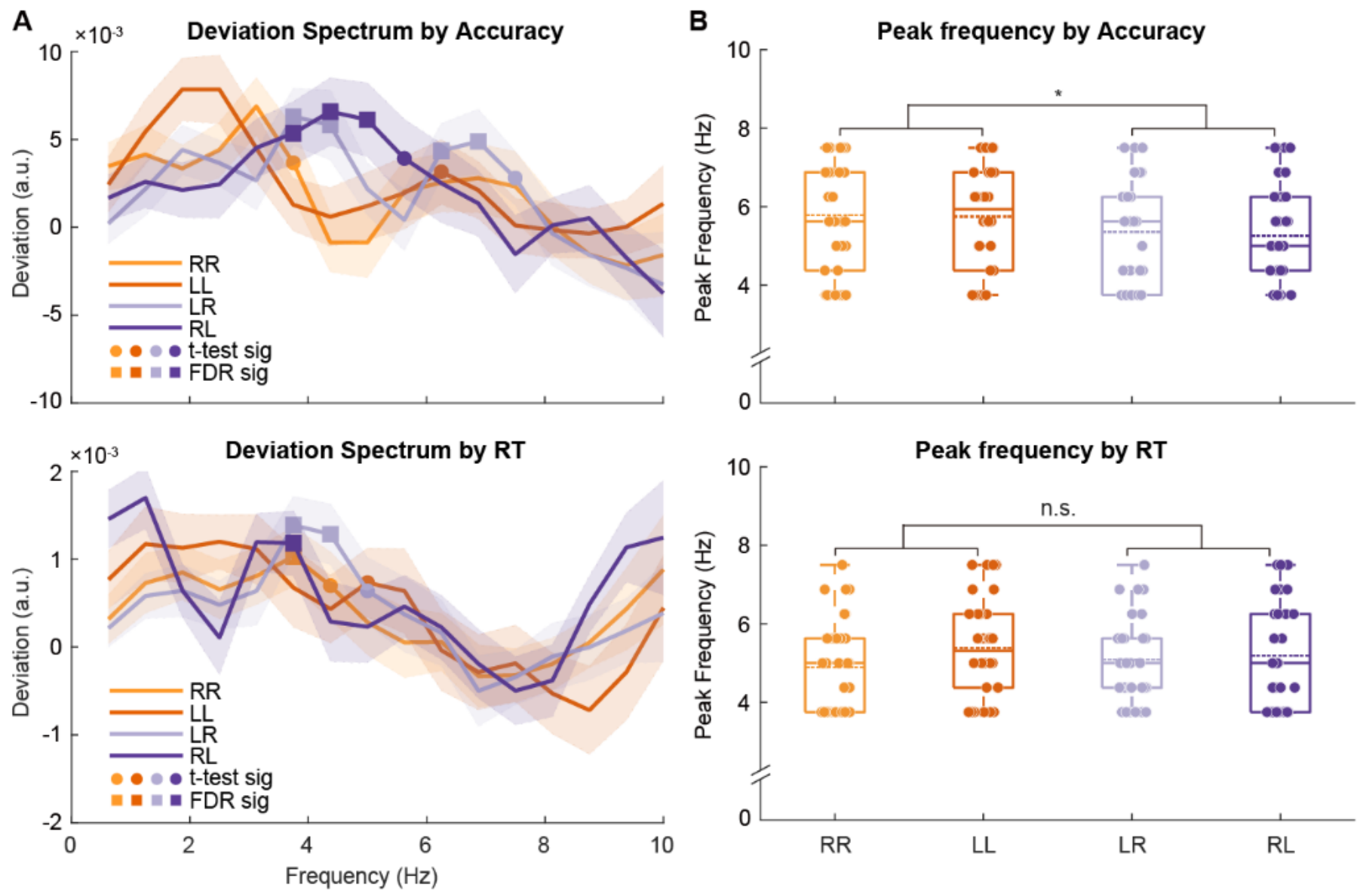

Figure 3. Results of Experiment 1 (n = 34 participants). (A) Group-mean deviation spectra for accuracy (top) and reaction time (bottom). Curves correspond to the four conditions (RR, LL, LR, RL); shaded areas indicate ± s.e.m. Filled circles mark frequencies significant by one-tailed one-sample t-tests across participants ($p < 0.05$); filled squares mark frequencies surviving Benjamini–Hochberg FDR correction across frequencies ($q = 0.05$). Statistical analysis was restricted to the theta-frequency range (3.75 - 7.50 Hz). (B) Distributions of individual theta peak frequencies for accuracy (top) and reaction time (bottom). Box plots show the median and interquartile range (whiskers, 1.5 × IQR); points indicate individual participants; solid horizontal lines indicate the mean peak frequency. * indicates $p < .05$; n.s. indicates not significant.

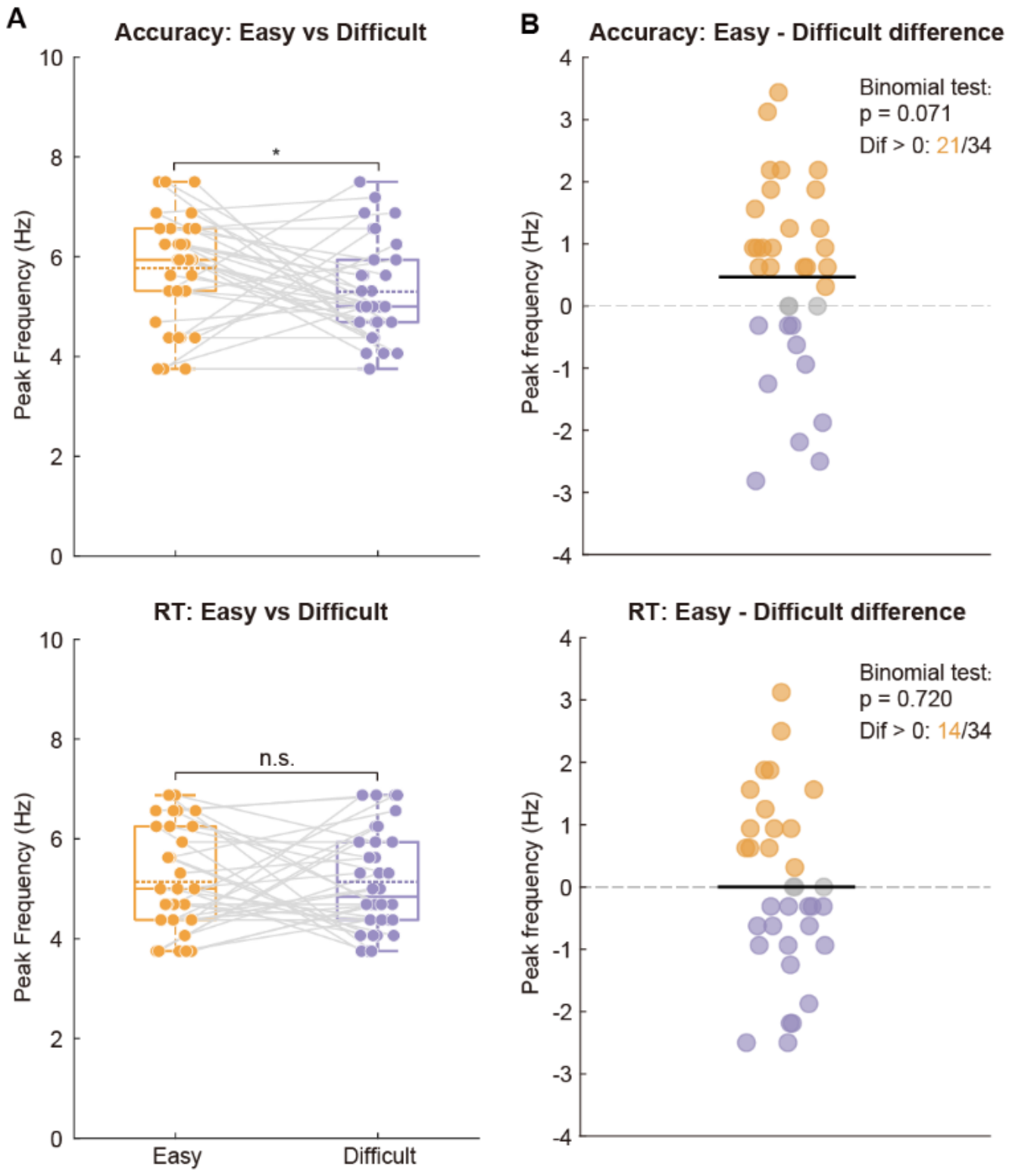

Figure 4. Peak frequency differences between easy and difficult rules (n = 34 participants). (A) Peak frequency for easy rules (RR and LL) and difficult rules (LR and RL), shown separately for accuracy-based estimates (top) and RT-based estimates (bottom). Dots represent individual participants, grey lines connect paired observations from the same participant, and boxplots show the group distributions. The dashed horizontal lines within each box indicate the condition means. (B) Participant-level easy-minus-difficult differences in peak frequency, shown separately for accuracy-based estimates (top) and RT-based estimates (bottom). Positive values indicate higher peak frequency for easy than difficult rules. The black horizontal line indicates the group mean difference, and the grey dashed line marks zero. Orange dots indicate participants with positive differences, purple dots indicate negative differences, and grey dots indicate zero differences. The inset reports the binomial test for the number of participants showing a positive difference. * indicates $p < .05$; n.s. indicates not significant.

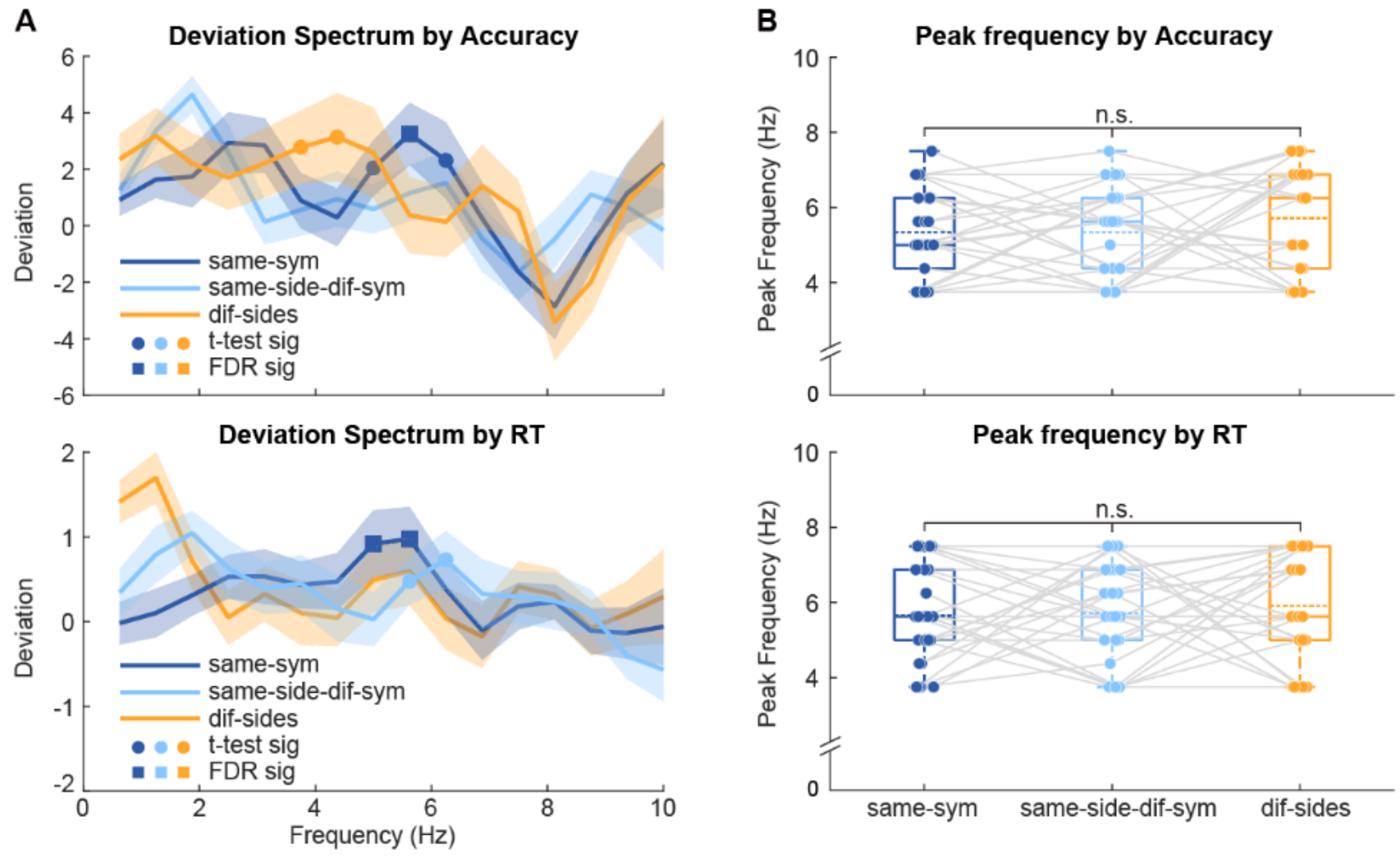

Figure 6. Results of Experiment 2 (n = 26 participants). (A) Group-mean deviation spectra for accuracy (top) and reaction time (bottom). Curves correspond to the three conditions (same-sym, same-side-dif-sym, and dif-sides); shaded areas indicate ± s.e.m. Filled circles mark frequencies significant by one-tailed one-sample t-tests across participants ($p < 0.05$); filled squares mark frequencies surviving Benjamini–Hochberg FDR correction across frequencies ($q = 0.05$). Statistical analysis was restricted to the theta-frequency range (3.75 - 7.50 Hz). (B) Distributions of individual theta peak frequencies for accuracy (top) and reaction time (bottom). Box plots show the median and interquartile range (whiskers, 1.5 × IQR); points indicate individual participants; solid horizontal lines indicate the mean peak frequency. n.s. indicates not significant.

Comment 7

Fig 3: maybe plot tests performed + outcomes for the significant difference in the figure for a better overview of the results.

Response

We have revised all relevant result figures to show the statistical outcomes more directly. Specifically, we added significance labels to the peak-frequency and behavioral-performance panels where condition comparisons were shown.

Comment 8

labels: SS, SSiDS, DSi: maybe more verbose labels for figures or different more verbose naming of conditions would make the article easier to read. Also, abbreviations like CCW, CW & others do not appear often, consider writing them out for legibility.

Response

We have revised the figure labels to reduce abbreviations and improve readability. Specifically, the condition labels SS, SSiDS, and DSi have been replaced with same-sym, same-side-dif-sym, and dif-sides, respectively. We also replaced CW and CCW with clockwise and counter-clockwise where they appeared in the figures.

Comment 9

It is not clear how much data was available in dataset #1, e.g. the number of trials is not mentioned. When I checked the original Senoussi et al. paper, I saw also that EEG data was collected. It would be good to mention, even if not analyzed here, to give a better picture of the structure of the dataset.

Response

We have clarified the Experiment 1 Methods by adding the approximate number of main-task trials before trial rejection and noting that the original dataset included behavioral, eye-tracking, and EEG recordings, although only the behavioral data were analyzed here. Specifically, we added (Page 5; Lines 158-160): "The main task comprised 5 - 8 blocks, depending on participant pace (participants with fewer missed trials completed more blocks), corresponding to approximately 448 - 704 trials before trial rejection."

We also added (Page 5; Lines 164-167): "The original dataset[28,31] included behavioral, eye-tracking, and EEG recordings, but the present reanalysis focused only on the behavioral data. Eye-tracking data were used for trial rejection and were aligned with the behavioral logs using EEG triggers (acquisition and processing details are provided in Senoussi et al.[28])."

Reviewer 3

General response

Thank you for all the insightful comments. We have revised the manuscript to clarify the interpretation of Experiment 2, add the suggested subgroup analysis, and improve several wording and citation issues. The main revisions are listed below:

(1) We clarified that the null frequency-shift result in Experiment 2 supports neither a task-difficulty account nor a cue ambiguity/entropy account.

(2) We added a post hoc subgroup analysis based on the apparent separation of different-sides peak-frequency estimates. This analysis is described in the Methods, reported in the Results, shown in the new Figure 7.

(3) We corrected "inter-trial intervals" to "inter-stimulus intervals" where appropriate.

(4) We revised the Results wording to avoid describing adjacent significant frequency bins as separate "peaks"; where appropriate, we now refer to significant frequency bins instead.

(5) We replaced the previous citation with the suggested references that more directly support the statement about neural oscillatory phase and perception/attention.

Comment 1

On my first readthrough, I misinterpreted the authors' conclusions regarding Experiment 2 as being in support of the cue ambiguity/entropy account. This was because they set the experiment up as testing competing difficulty versus ambiguity/entropy hypotheses, and when there were no differences, they concluded there were no difficulty effects. As the ambiguity hypothesis was not discussed any further, I took this at first to be the implicit winning hypothesis. I think the manuscript would be clearer if the authors also discussed the ambiguity/entropy account and stated for the reader's benefit that this account was also not supported.

Response

We have clarified the interpretation of Experiment 2 in the Results section. The revised text now makes explicit that the absence of a difficulty-related frequency shift should not be read as support for the cue ambiguity/entropy account: "Collectively, this new dataset[32] does not provide clear evidence for systematic modulation of the behavioral theta peak. Thus, the results do not provide clear support for either a task-difficulty account or a cue ambiguity/entropy account."

Comment 2

In figure 4B, the data for the DSi condition looks quite bimodal for both measures. It might be fruitful to perform an exploratory analysis where you split the data between the high and low peak-frequency clusters and look at whether there might be subsets in the data that are showing different patterns.

Response

We thank the reviewer for this helpful suggestion. Following this comment, we added a post hoc subgroup analysis to examine whether the apparent bimodal distribution of peak-frequency estimates in the different-sides condition reflected broader participant-level differences. Participants were assigned to lower- and higher-frequency subgroups, and we compared these groups in peak frequency, behavioral performance, and DDM-derived parameters.

We have described this analysis in the Methods section (Page 8; Lines 289–298): "**Subgroup analysis based on different-sides peak frequency.** To examine the gap in the individual peak-frequency estimates in the dif-sides condition, we conducted a post hoc subgroup analysis. Participants were assigned to either the low- or high-frequency subgroup based on the median frequency bin in the accuracy-based dif-sides peak-frequency distribution. We then examined whether these subgroups differed in peak frequency, behavioral performance, and diffusion decision model (DDM) parameters, including boundary separation, drift rate, and non-decision time. DDM parameters were estimated using the EZ-diffusion method[33] separately for each participant and condition, based on the proportion of correct responses, mean RT for correct trials, and RT variance for correct trials. Trials with RTs ≤ 0.2 s or ≥ 3 s were excluded before parameter estimation.

"

The results are reported in the Results section and shown in the new Figure 7 (Page 11-12; Lines 439 – 487): "**Subgroup patterns based on different-sides peak frequency.** Because individual peak-frequency estimates in the dif-sides condition (Fig. 6B, top) appeared to cluster into low- and high-frequency ranges, we examined whether this separation was associated with differences in behavior or DDM-derived parameters. Participants were assigned to low- and high-frequency subgroups according to whether their dif-sides peak frequency fell below or above the median value of 5.62 Hz (low-frequency subgroup: n = 12; high-frequency subgroup: n = 14; Fig. 7A), and subgroup effects were then tested across conditions using the linear mixed-effects models (see Methods for details).

As expected, peak frequency showed a significant subgroup effect (Type-III ANOVA with Satterthwaite df: $F_{(1, 26)} = 19.49$, $p < .001$, $\eta_p^2 = .43$, 95% CI [.14, .63]) and a significant subgroup × condition interaction ($F_{(2, 52)} = 13.36$, $p < .001$, $\eta_p^2 = .34$, 95% CI [.13, .50]), while the main effect of condition was not significant ($F_{(2, 52)} = 0.83$, $p = .442$, $\eta_p^2 = .03$, 95% CI [.00, .15]; Fig. 7B, top left). Follow-up Welch independent-samples t-tests confirmed that the low- and high-frequency subgroups differed in the dif-sides condition ($t_{(21.20)} = -12.78$, $p < .001$, Cohen's $d = -5.07$, 95% CI [−6.76, −3.35]), but not in the same-sym ($t_{(23.69)} = -0.52$, $p = .609$, Cohen's $d = -0.20$, 95% CI [−0.96, 0.57]) or same-side-dif-sym conditions ($t_{(23.48)} = -0.53$, $p = .604$, Cohen's $d = -0.20$, 95% CI [−0.97, 0.56]).

We then asked whether this subgroup split was reflected in behavioral performance. For accuracy, the linear mixed-effects model showed a significant condition effect ($F_{(2, 52)} = 17.32$, $p < .001$, $\eta_p^2 = .40$, 95% CI [.19, .56]) and a subgroup × condition interaction ($F_{(2, 52)} = 3.25$, $p = .047$, $\eta_p^2 = .11$, 95% CI [.00, .27]), but no main effect of subgroup ($F_{(1, 26)} = 1.08$, $p = .309$, $\eta_p^2 = .04$, 95% CI [.00, .26]; Fig. 7B, middle left). This pattern suggests that the high-frequency subgroup showed a stronger accuracy decrease in the dif-sides condition, although the two subgroups did not differ significantly within any single condition (same-sym: $t_{(17.97)} = -1.68$, $p = .111$, Cohen's $d = -0.67$, 95% CI [−1.48, 0.15]; same-side-dif-sym: $t_{(20.52)} = -1.34$, $p = .194$, Cohen's $d = -0.53$, 95% CI [−1.32, 0.27]; dif-sides: $t_{(23.84)} = 0.49$, $p = .626$, Cohen's $d = 0.19$, 95% CI [−0.58, 0.96]). RT showed only a condition effect ($F_{(2, 52)} = 19.69$, $p < .001$, $\eta_p^2 = .43$, 95% CI [.22, .58]), with no subgroup effect ($F_{(1, 26)} = 0.04$, $p = .838$, $\eta_p^2 = .00$, 95% CI [.00, .13]) or subgroup × condition interaction ($F_{(2, 52)} = 0.10$, $p = .908$, $\eta_p^2 = .00$, 95% CI [.00, .05]; Fig. 7B, bottom left).

For the DDM parameters, boundary separation did not show significant effects of subgroup ($F_{(1, 26)} = 0.42$, $p = .521$, $\eta_p^2 = .02$, 95% CI [.00, .21]), condition ($F_{(2, 52)} = 1.50$, $p = .232$, $\eta_p^2 = .05$, 95% CI [.00, .19]), or their interaction ($F_{(2, 52)} = 0.71$, $p = .498$, $\eta_p^2 = .03$, 95% CI [.00, .14]; Fig. 7B, top right). Drift rate showed a significant condition effect ($F_{(2, 52)} = 19.66$, $p < .001$, $\eta_p^2 = .43$, 95% CI [.22, .58]), and visually followed a similar pattern to accuracy, although neither the subgroup effect ($F_{(1, 26)} = 1.64$, $p = .212$, $\eta_p^2 = .06$, 95% CI [.00, .29]) nor the subgroup × condition interaction reached significance ($F_{(2, 52)} = 3.12$, $p = .052$, $\eta_p^2 = .11$, 95% CI [.00, .27]; Fig. 7B, middle right). Non-decision time showed a condition effect ($F_{(2, 52)} = 4.96$, $p = .011$, $\eta_p^2 = .16$,

95% CI [.01, .33]), but no subgroup effect ($F_{(1, 26)}$ = 0.33, p = .572, η_p^2 = .01, 95% CI [.00, .20]) or subgroup × condition interaction ($F_{(2, 52)}$ = 0.33, p = .717, η_p^2 = .01, 95% CI [.00, .10]; Fig. 7B, bottom right).

Overall, the subgroup split was most clearly reflected in the dif-sides peak-frequency estimates themselves. Beyond this defining difference, there was limited evidence for systematic subgroup differences in behavior or DDM parameters, although the high-frequency subgroup showed a stronger dif-sides-related decrease in accuracy."

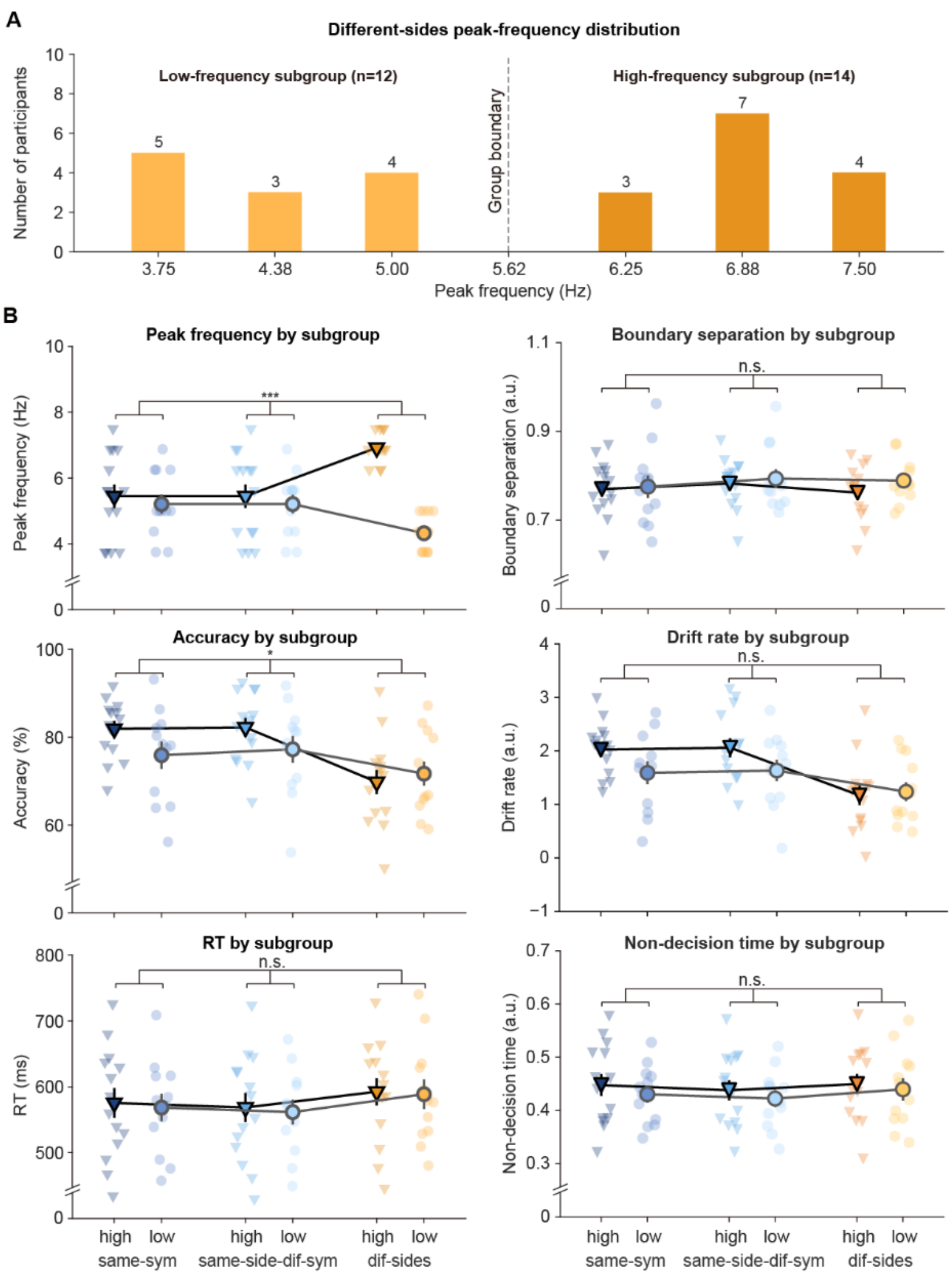

Figure 7. Peak-frequency, behavioral, and DDM measures in the low-frequency subgroup (n = 12 participants) and high-frequency subgroup (n = 14 participants). (A) Distribution of individual peak-frequency estimates in the different-sides condition. Participants were assigned to the low- or high-frequency subgroup according to whether their different-sides peak frequency fell below or above 5.62 Hz, marked by the dashed vertical line. Bars indicate the number of participants in each peak-frequency bin. (B) Peak-frequency, behavioral, and DDM measures for the low- and high-frequency subgroups across conditions. Panels show peak frequency (top left), boundary separation (top right), accuracy (middle left), drift rate (middle right), response time (RT; bottom

left), and non-decision time (bottom right). Individual transparent points indicate participants. Triangles indicate the high-frequency subgroup, and circles indicate the low-frequency subgroup. Larger black triangles and grey circles connected by lines indicate subgroup means across conditions. *** indicates $p < .001$; * indicates $p < .05$; n.s. indicates not significant.

The analysis showed that the separation was clearest for the different-sides peak-frequency estimates themselves. However, it did not reveal a consistent behavioral or DDM profile that would support a stable subgroup distinction. Thus, we treat the apparent bimodality as evidence for heterogeneity in peak-frequency estimates, rather than as evidence for two mechanistically distinct participant groups.

Comment 3

Lines 122-123: “inter-trial intervals” should be “inter-stimulus intervals”

Response

Thank you for pointing this out. We have corrected this wording and replaced “inter-trial intervals” with “inter-stimulus intervals” in the revised manuscript.

Comment 4

Throughout the Results, cases with multiple significant frequencies are described as multiple “significant peaks” when, from the plots, they appear in each case to be multiple points spanning a single peak. Perhaps this could be reworded?

Response

We have revised the wording in the Results section to avoid describing adjacent significant frequencies as separate “peaks.” Where appropriate, we now refer to significant frequency bins instead.

Comment 5

Line 45: reference 13 (Fiebelkorn et al., 2013, Current Biology) is a behavioural oscillations study. As they did not measure brain activity, their conclusions cannot support the relationship between instantaneous phase and perception/attention.

Lines 44-45: Instead of the above, may I shamelessly suggest a citation of my own work in this section:

o Attention & perception: Harris, A. M., Dux, P. E., & Mattingley, J. B. (2018). Detecting unattended stimuli depends on the phase of prestimulus neural oscillations. Journal of Neuroscience, 38(12), 3092-3101.

o Perception: Williams, J. G., Harrison, W. J., Beale, H. A., Mattingley, J. B., & Harris, A. M. (2024). Effects of neural oscillation power and phase on discrimination performance in a visual tilt illusion. Current Biology, 34(8), 1801-1809.

Response

Thank you for catching this and for the suggested references. We have replaced Fiebelkorn et

al. (2013) with Harris et al. (2018) and Williams et al. (2024), as suggested.

Reviewer 1

Comment 1

The authors have addressed many of my concerns and the manuscript is much improved on several levels. At this time - the only remaining point I noticed upon re-reading the revised manuscript is the following sentence in the abstract: " However, identifying such a behavioral signature has remained controversial: evidence for behavioral oscillations is not yet firmly established, and aperiodic components can be misidentified as rhythmic". I think that this paper makes an interesting a solid contribution to the literature - but it isn't the first to account for aperiodicity in behavioural oscillations. For example, if I am not mistaken, aperiodic components of the spectra were considered by, e.g., Re et al., 2023 J Con Neuro in their revised analysis of accuracy time courses. It ok to indicate there is controversy and room for a closer analytic consideration. But it probably is slightly overstated to say that evidence for behavioural oscillations is not yet firmly established -- and for this particular reason. It is a small thing - and mostly relates to the culture of advancing knowledge rather than of true essence.

Response

We thank the reviewer for this helpful comment. We agree that the previous wording was somewhat too strong and did not sufficiently acknowledge prior work on behavioral oscillations and aperiodic components. We have therefore revised the Abstract to emphasize that identifying behavioral rhythmicity remains analytically challenging and debated, rather than suggesting that the evidence is broadly not established (Page 1; Lines 11-16): "However, identifying such behavioral signatures remains challenging and continues to motivate debate, particularly because aperiodic temporal structure can sometimes be mistaken for rhythmic activity. To assess behavioral rhythmicity while controlling for aperiodic dynamics, we applied a state-of-the-art autoregressive modelling approach to two datasets: one published dataset (final sample: n = 34), and a new dataset (final sample: n = 26) collected with an adapted dense-sampling design."

Reviewer 2

We thank the reviewer for their positive assessment and are pleased that our responses addressed their concerns.

Reviewer 3

We thank the reviewer for their helpful feedback and for acknowledging the additional analyses.
