# Supplementary material for: Robust evidence for theta-band rhythmicity in behavior across two dense-sampling datasets
Source: Commun Psychol. 2026 Jul 7;4:155. doi: 10.1038/s44271-026-00500-0 (PMC13728126; doi:10.1038/s44271-026-00500-0)
Supplement: Supplementary file 2 [file 44271_2026_500_MOESM2_ESM.pdf]

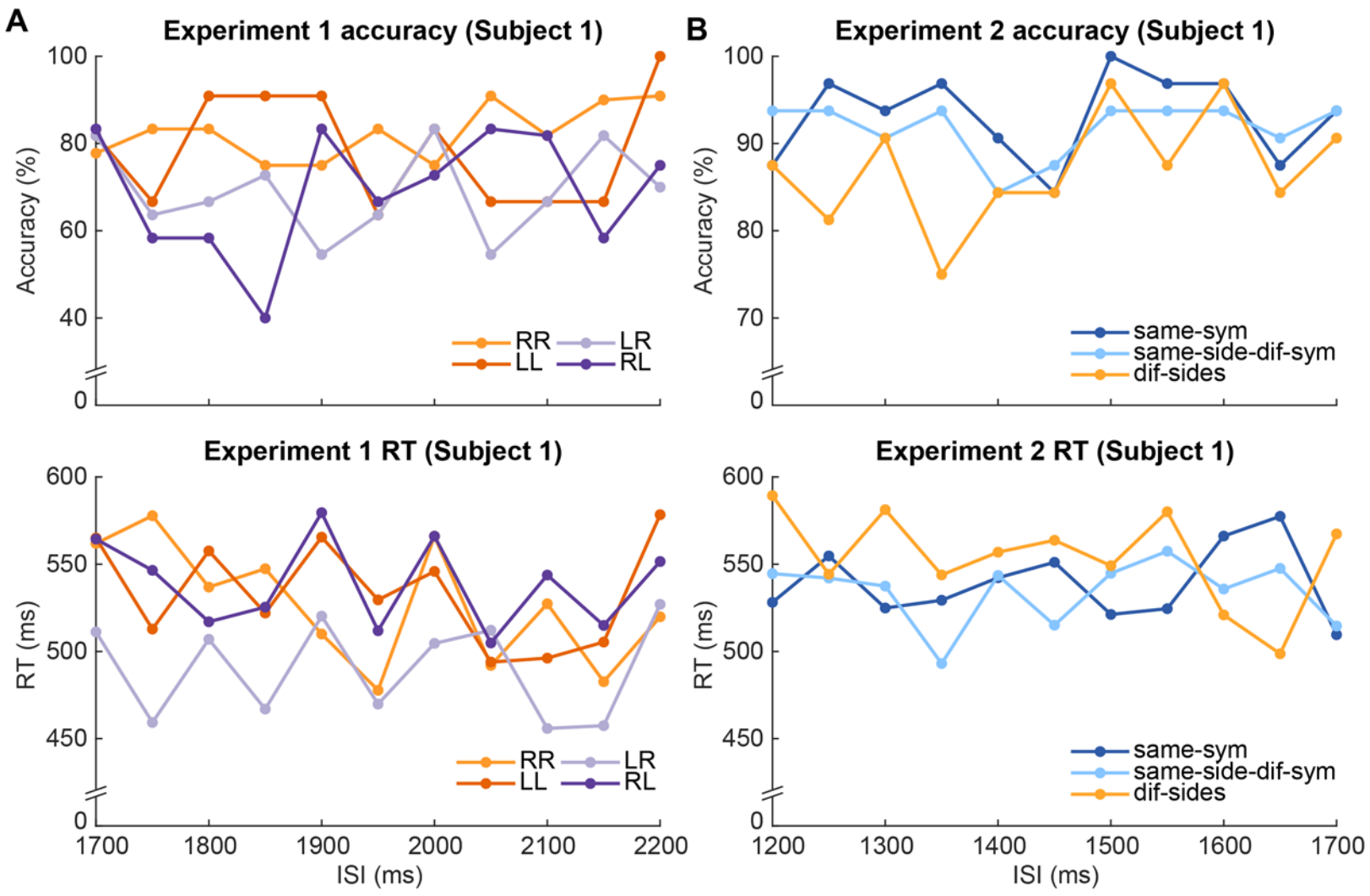

Supplementary Figure 1. Example single-participant behavioral time courses. (A) Accuracy and response time (RT) time courses across inter-stimulus intervals (ISIs) for Subject 1 in Experiment 1. (B) Accuracy and RT time courses across ISIs for Subject 1 in Experiment 2. These time courses illustrate the behavioral data entered into the autoregressive-surrogate spectral analysis.

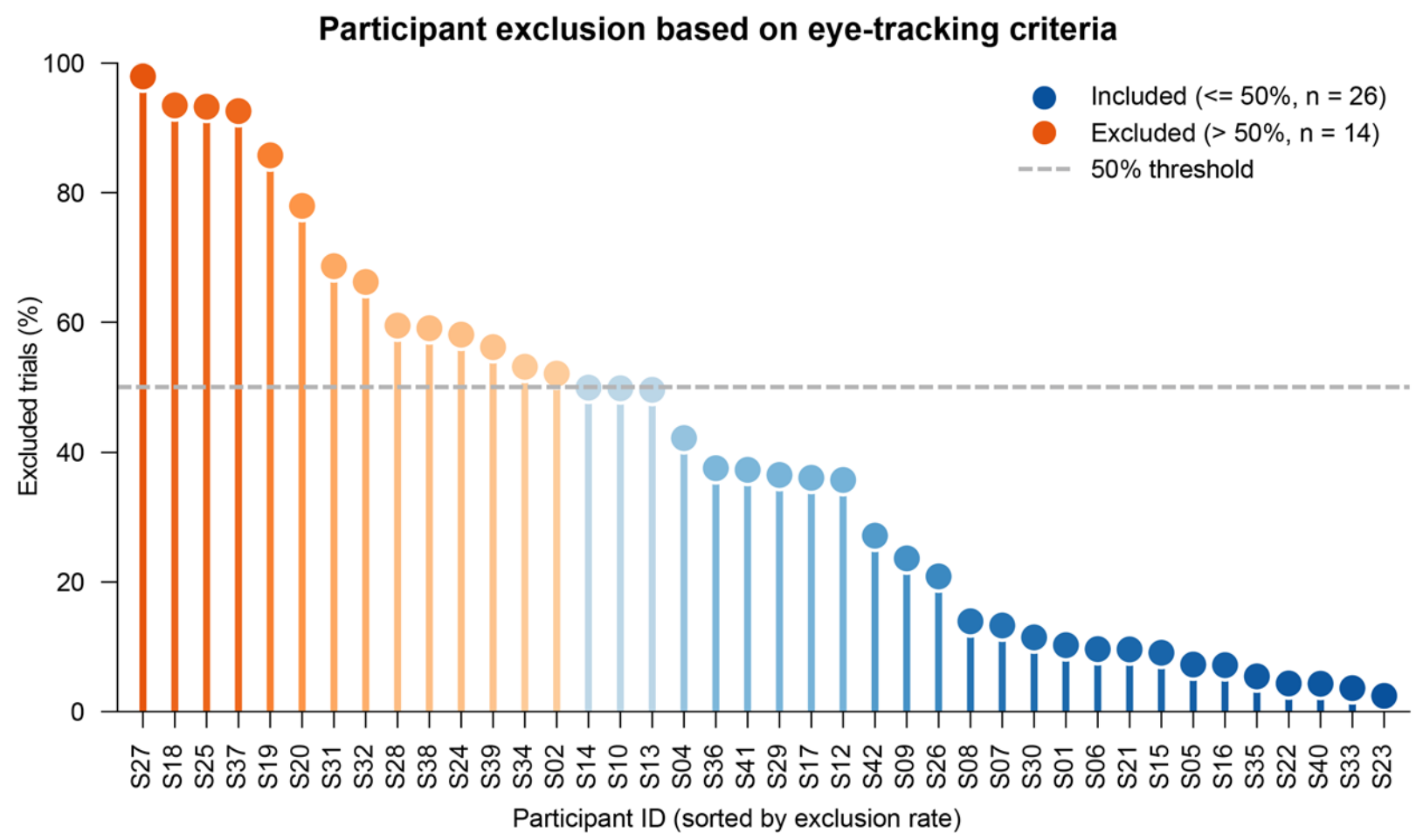

Supplementary Figure 2. Eye-tracking-related trial exclusion rate in Experiment 2. Each point shows the subject-level mean percentage of trials excluded by the eye-tracking criteria across sessions. Participants are sorted from highest to lowest exclusion rate. Orange points indicate participants excluded by the predefined criterion (>50% trials rejected), and blue points indicate participants retained for analysis (≤50% trials rejected). The dashed line marks the 50% exclusion threshold.

Supplementary Table 1.

Number of participants showing at least one positive theta-band deviation.

Experiment	Measure	Condition	Rhythmic Count
Experiment 1	Accuracy	RR	33/34
		LL	34/34
		LR	34/34
		RL	34/34
	Response time	RR	33/34
		LL	33/34
		LR	34/34
		RL	34/34
Experiment 2	Accuracy	same-sym	26/26
		same-side-dif-sym	25/26
		dif-sides	24/26
	Response time	same-sym	25/26
		same-side-dif-sym	26/26
		dif-sides	25/26

Note. Participants were counted as showing behavioral rhythmicity if their deviation spectrum showed at least one positive value in the theta range (3.75–7.50 Hz). Counts indicate the number of participants meeting this criterion out of the total number of participants.

Supplementary Table 2.

Full frequency-wise statistics for accuracy deviation spectra in Experiment 1.

Condition	Frequency (Hz)	$t_{(df)}$	*p*	p_{FDR}	Cohen's d_z	95% CI for d_z
RR	3.75	$t_{(33)}$ = 1.82	.039	.178	0.31	[-0.05, 0.67]
	4.38	$t_{(33)}$ = -0.52	.697	.697	-0.09	[-0.44, 0.26]
	5.00	$t_{(33)}$ = -0.43	.664	.697	-0.07	[-0.42, 0.28]
	5.62	$t_{(33)}$ = 1.18	.123	.178	0.20	[-0.15, 0.56]
	6.25	$t_{(33)}$ = 1.51	.070	.178	0.26	[-0.10, 0.61]
	6.88	$t_{(33)}$ = 1.42	.083	.178	0.24	[-0.11, 0.60]
	7.50	$t_{(33)}$ = 1.16	.127	.178	0.20	[-0.15, 0.55]
LL	3.75	$t_{(33)}$ = 0.87	.195	.340	0.15	[-0.20, 0.50]
	4.38	$t_{(33)}$ = 0.36	.359	.419	0.06	[-0.29, 0.41]
	5.00	$t_{(33)}$ = 0.62	.269	.376	0.11	[-0.24, 0.46]
	5.62	$t_{(33)}$ = 0.96	.172	.340	0.16	[-0.19, 0.52]
	6.25	$t_{(33)}$ = 1.70	.049	.340	0.29	[-0.06, 0.65]
	6.88	$t_{(33)}$ = 1.28	.104	.340	0.22	[-0.13, 0.57]
	7.50	$t_{(33)}$ = 0.08	.468	.468	0.01	[-0.34, 0.36]
LR	3.75	$t_{(33)}$ = 3.89	<.001	.002	0.67	[0.28, 1.05]
	4.38	$t_{(33)}$ = 2.92	.003	.010	0.50	[0.13, 0.87]
	5.00	$t_{(33)}$ = 1.51	.071	.082	0.26	[-0.10, 0.61]
	5.62	$t_{(33)}$ = 0.25	.404	.404	0.04	[-0.31, 0.39]
	6.25	$t_{(33)}$ = 2.81	.004	.010	0.48	[0.11, 0.85]
	6.88	$t_{(33)}$ = 2.67	.006	.010	0.46	[0.09, 0.83]
	7.50	$t_{(33)}$ = 1.84	.038	.053	0.32	[-0.04, 0.67]
RL	3.75	$t_{(33)}$ = 2.96	.003	.007	0.51	[0.14, 0.88]
	4.38	$t_{(33)}$ = 3.40	<.001	.006	0.58	[0.20, 0.96]
	5.00	$t_{(33)}$ = 2.92	.003	.007	0.50	[0.13, 0.87]
	5.62	$t_{(33)}$ = 1.76	.044	.077	0.30	[-0.05, 0.66]
	6.25	$t_{(33)}$ = 1.12	.136	.191	0.19	[-0.16, 0.54]
	6.88	$t_{(33)}$ = 0.73	.234	.273	0.13	[-0.22, 0.48]
	7.50	$t_{(33)}$ = -0.68	.748	.748	-0.12	[-0.47, 0.23]

Note. Statistics are shown for all frequency bins within the analysed theta range (3.75–7.50 Hz). For each condition and frequency bin, one-tailed one-sample t-tests were performed against zero on participant-level deviation values. The *p* column reports uncorrected p-values, whereas p_{FDR} reports *p* values corrected within each condition using the Benjamini-Hochberg false discovery rate (FDR) procedure. Cohen's d_z is reported as the effect size, with 95% confidence intervals (CI).

Supplementary Table 3.

Full frequency-wise statistics for RT deviation spectra in Experiment 1.

Condition	Frequency (Hz)	$t_{(df)}$	*p*	p_{FDR}	Cohen's d_z	95% CI for d_z
RR	3.75	$t_{(33)}$ = 3.01	.002	.017	0.52	[0.14, 0.89]
	4.38	$t_{(33)}$ = 1.74	.046	.160	0.30	[-0.06, 0.65]
	5.00	$t_{(33)}$ = 0.75	.230	.536	0.13	[-0.22, 0.48]
	5.62	$t_{(33)}$ = 0.13	.447	.626	0.02	[-0.33, 0.37]
	6.25	$t_{(33)}$ = 0.21	.419	.626	0.04	[-0.31, 0.38]
	6.88	$t_{(33)}$ = -0.85	.800	.824	-0.15	[-0.50, 0.20]
	7.50	$t_{(33)}$ = -0.94	.824	.824	-0.16	[-0.51, 0.19]
LL	3.75	$t_{(33)}$ = 1.48	.074	.206	0.25	[-0.10, 0.61]
	4.38	$t_{(33)}$ = 1.11	.138	.241	0.19	[-0.16, 0.54]
	5.00	$t_{(33)}$ = 1.87	.035	.206	0.32	[-0.04, 0.68]
	5.62	$t_{(33)}$ = 1.38	.088	.206	0.24	[-0.12, 0.59]
	6.25	$t_{(33)}$ = 0.01	.497	.695	0.00	[-0.35, 0.35]
	6.88	$t_{(33)}$ = -0.62	.730	.730	-0.11	[-0.46, 0.24]
	7.50	$t_{(33)}$ = -0.41	.659	.730	-0.07	[-0.42, 0.28]
LR	3.75	$t_{(33)}$ = 4.18	<.001	<.001	0.72	[0.32, 1.11]
	4.38	$t_{(33)}$ = 3.74	<.001	.001	0.64	[0.26, 1.03]
	5.00	$t_{(33)}$ = 1.73	.046	.108	0.30	[-0.06, 0.65]
	5.62	$t_{(33)}$ = 0.86	.198	.346	0.15	[-0.20, 0.50]
	6.25	$t_{(33)}$ = 0.37	.356	.498	0.06	[-0.29, 0.41]
	6.88	$t_{(33)}$ = -1.24	.889	.889	-0.21	[-0.57, 0.14]
	7.50	$t_{(33)}$ = -1.11	.862	.889	-0.19	[-0.54, 0.16]
RL	3.75	$t_{(33)}$ = 3.11	.002	.013	0.53	[0.16, 0.91]
	4.38	$t_{(33)}$ = 0.66	.257	.435	0.11	[-0.24, 0.46]
	5.00	$t_{(33)}$ = 0.61	.273	.435	0.10	[-0.25, 0.45]
	5.62	$t_{(33)}$ = 1.23	.113	.396	0.21	[-0.14, 0.56]
	6.25	$t_{(33)}$ = 0.50	.311	.435	0.09	[-0.26, 0.44]
	6.88	$t_{(33)}$ = -0.53	.701	.818	-0.09	[-0.44, 0.26]
	7.50	$t_{(33)}$ = -1.33	.904	.904	-0.23	[-0.58, 0.13]

Note. Statistics are shown for all frequency bins within the analysed theta range (3.75–7.50 Hz). For each condition and frequency bin, one-tailed one-sample t-tests were performed against zero on participant-level deviation values. The *p* column reports uncorrected p-values, whereas p_{FDR} reports *p* values corrected within each condition using the Benjamini-Hochberg false discovery rate (FDR) procedure. Cohen's d_z is reported as the effect size, with 95% confidence intervals (CI).

Supplementary Table 4.

Full frequency-wise statistics for accuracy deviation spectra in Experiment 2.

Condition	Frequency (Hz)	$t_{(df)}$	*p*	p_{FDR}	Cohen's d_z	95% CI for d_z
same-sym	3.75	$t_{(25)}$ = 0.91	.185	.324	0.18	[-0.23, 0.59]
	4.38	$t_{(25)}$ = 0.29	.388	.490	0.06	[-0.35, 0.46]
	5.00	$t_{(25)}$ = 2.09	.023	.082	0.41	[-0.01, 0.83]
	5.62	$t_{(25)}$ = 2.96	.003	.023	0.58	[0.14, 1.02]
	6.25	$t_{(25)}$ = 1.71	.0497	.116	0.34	[-0.08, 0.75]
	6.88	$t_{(25)}$ = 0.20	.420	.490	0.04	[-0.36, 0.44]
	7.50	$t_{(25)}$ = -1.56	.934	.934	-0.31	[-0.72, 0.11]
same-	3.75	$t_{(25)}$ = 0.62	.271	.380	0.12	[-0.28, 0.53]
side-	4.38	$t_{(25)}$ = 0.96	.172	.380	0.19	[-0.22, 0.60]
dif-sym	5.00	$t_{(25)}$ = 0.68	.251	.380	0.13	[-0.27, 0.54]
	5.62	$t_{(25)}$ = 1.16	.128	.380	0.23	[-0.18, 0.64]
	6.25	$t_{(25)}$ = 1.36	.093	.380	0.27	[-0.14, 0.68]
	6.88	$t_{(25)}$ = -0.39	.649	.757	-0.08	[-0.48, 0.33]
	7.50	$t_{(25)}$ = -1.65	.945	.945	-0.32	[-0.74, 0.09]
dif-sides	3.75	$t_{(25)}$ = 2.16	.020	.096	0.42	[0.00, 0.85]
	4.38	$t_{(25)}$ = 2.02	.027	.096	0.40	[-0.02, 0.82]
	5.00	$t_{(25)}$ = 1.62	.059	.137	0.32	[-0.10, 0.73]
	5.62	$t_{(25)}$ = 0.27	.394	.456	0.05	[-0.35, 0.46]
	6.25	$t_{(25)}$ = 0.11	.456	.456	0.02	[-0.38, 0.43]
	6.88	$t_{(25)}$ = 0.97	.171	.300	0.19	[-0.22, 0.60]
	7.50	$t_{(25)}$ = 0.47	.322	.451	0.09	[-0.31, 0.50]

Note. Statistics are shown for all frequency bins within the analysed theta range (3.75–7.50 Hz). For each condition and frequency bin, one-tailed one-sample t-tests were performed against zero on participant-level deviation values. The *p* column reports uncorrected p-values, whereas p_{FDR} reports *p* values corrected within each condition using the Benjamini-Hochberg false discovery rate (FDR) procedure. Cohen's d_z is reported as the effect size, with 95% confidence intervals (CI).

Supplementary Table 5.

Full frequency-wise statistics for RT deviation spectra in Experiment 2.

Condition	Frequency (Hz)	$t_{(df)}$	p	p_{FDR}	Cohen's d_z	95% CI for d_z
same-sym	3.75	$t_{(25)}$ = 1.57	.064	.146	0.31	[-0.11, 0.72]
	4.38	$t_{(25)}$ = 1.42	.084	.146	0.28	[-0.13, 0.69]
	5.00	$t_{(25)}$ = 2.33	.014	.049	0.46	[0.03, 0.88]
	5.62	$t_{(25)}$ = 2.57	.008	.049	0.50	[0.07, 0.93]
	6.25	$t_{(25)}$ = 1.22	.117	.164	0.24	[-0.17, 0.65]
	6.88	$t_{(25)}$ = -0.30	.615	.615	-0.06	[-0.46, 0.35]
	7.50	$t_{(25)}$ = 0.66	.258	.301	0.13	[-0.28, 0.53]
same-side-dif-sym	3.75	$t_{(25)}$ = 1.35	.095	.222	0.26	[-0.15, 0.67]
	4.38	$t_{(25)}$ = 0.40	.345	.402	0.08	[-0.33, 0.48]
	5.00	$t_{(25)}$ = 0.09	.464	.464	0.02	[-0.39, 0.42]
	5.62	$t_{(25)}$ = 1.74	.047	.165	0.34	[-0.07, 0.76]
	6.25	$t_{(25)}$ = 2.15	.021	.144	0.42	[0.00, 0.84]
	6.88	$t_{(25)}$ = 0.81	.212	.297	0.16	[-0.25, 0.57]
	7.50	$t_{(25)}$ = 0.96	.174	.297	0.19	[-0.22, 0.60]
dif-sides	3.75	$t_{(25)}$ = 0.27	.393	.533	0.05	[-0.35, 0.46]
	4.38	$t_{(25)}$ = 0.14	.446	.533	0.03	[-0.38, 0.43]
	5.00	$t_{(25)}$ = 1.28	.106	.247	0.25	[-0.16, 0.66]
	5.62	$t_{(25)}$ = 1.49	.074	.247	0.29	[-0.12, 0.71]
	6.25	$t_{(25)}$ = 0.11	.457	.533	0.02	[-0.38, 0.43]
	6.88	$t_{(25)}$ = -0.47	.677	.677	-0.09	[-0.50, 0.31]
	7.50	$t_{(25)}$ = 1.39	.089	.247	0.27	[-0.14, 0.68]

Note. Statistics are shown for all frequency bins within the analysed theta range (3.75–7.50 Hz). For each condition and frequency bin, one-tailed one-sample t-tests were performed against zero on participant-level deviation values. The p column reports uncorrected p-values, whereas p_{FDR} reports p values corrected within each condition using the Benjamini-Hochberg false discovery rate (FDR) procedure. Cohen's d_z is reported as the effect size, with 95% confidence intervals (CI).
